# Isolation and Characterization of Two New Metabolites from the Sponge-Derived Fungus *Aspergillus* sp. LS34 by OSMAC Approach

**DOI:** 10.3390/md17050283

**Published:** 2019-05-11

**Authors:** Wei Li, Lijian Ding, Ning Wang, Jianzhou Xu, Weiyan Zhang, Bin Zhang, Shan He, Bin Wu, Haixiao Jin

**Affiliations:** 1 Li Dak Sum Yip Yio Chin Kenneth Li Marine Biopharmaceutical Research Center, College of Food and Pharmaceutical Sciences, Ningbo University, Ningbo 315800, China; 15058882124@163.com (W.L.); wangning2@nbu.edu.cn (N.W.); 15669221063@163.com (J.X.); zhangweiyan@nbu.edu.cn (W.Z.); zhangbin1@nbu.edu.cn (B.Z.); heshan@nbu.edu.cn (S.H.); 2 Ocean College, Zhejiang University, Hangzhou 310058, China; wubin@zju.edu.cn

**Keywords:** OSMAC, fungal natural product, antibacterial, cytotoxic

## Abstract

The application of an OSMAC (One Strain-Many Compounds) approach on the sponge-derived fungus *Aspergillus* sp. LS34, using two different media including solid rice medium and potato dextrose broth (PDB) resulted in the isolation and identification of two new compounds, named asperspin A (**1**) and asperther A (**2**) along with seven known compounds **3**–**9**. Compounds **1**–**5** were detected in fungal extracts from rice medium, while compounds **6**–**9** were isolated from PDB medium. Their structures were unambiguously characterized by HRESIMS and NMR spectroscopic data. The growth inhibitory activity of these compounds against four pathogenic bacteria (*Vibrio parahaemolyticus*, *Vibrio harveyi*, *Escherichia coli*, and *Staphylococcus aureus*) were evaluated. All the compounds were also tested for their cytotoxicity against seven cancer cell lines, including CCRF-CEM, K562, BGC823, AGS, HCT-116, MDA-MB-453, and COR-L23. Among them, compound **9** showed strong activity against CCRF-CEM and K562 cells with IC_50_ values of 1.22 ± 0.05 µM and 10.58 ± 0.19 µM, respectively. Notably, compound **7** also showed pronounced activity against *S. aureus* with an MIC value of 3.54 µM.

## 1. Introduction

**Figure 1:**
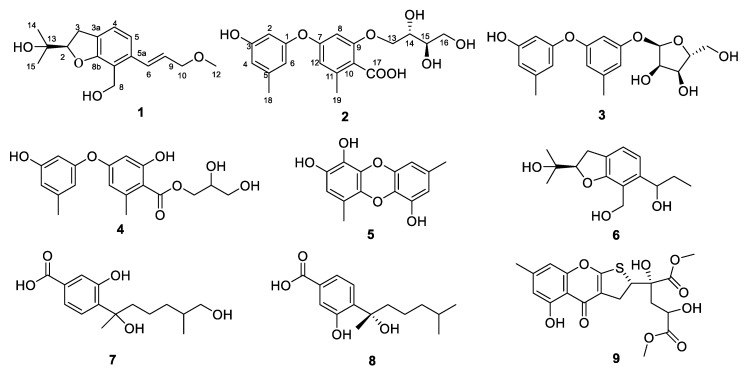
Structures of compounds **1**–**9**.

Marine microorganisms, in particular marine-derived fungi, have proven to be a promising producer of biologically active secondary metabolites for new chemicals in drug discovery [1,2,3,4,5]. Whole genome sequencing of fungi has showed the existence of silent pathways, which are not expressed under standard culture conditions [6]. However, changing culture conditions can activate potentially silent gene clusters thereby increasing the variety of secondary metabolites [7]. The OSMAC (One Strain−Many Compounds) approach has been shown to be a powerful strategy for triggering silent gene clusters by changing different parameters [8]. For example, one successful application was the isolation of novel pimarane diterpenoids from Arctic soil-derived fungus *Eutypella* sp. D-1, via the modification of the culture medium [9]. In the past decades, sponge-derived fungi in the genus *Aspergillus* have produced various metabolites, which have displayed biological and pharmacological activities such as antiviral [10], antibacterial [11], antitumor [12], and anti-inflammatory [13,14]. In this work, the OSMAC approach was employed for comparison of the metabolic profiles of the sponge-derived fungus *Aspergillus* sp. LS34 cultured on PDB medium and solid rice medium, respectively. The respective EtOAc extracts obtained from two cultures were analyzed by HPLC, revealing interesting variations in their secondary metabolites. The chromatographic study of these extracts led to two new compounds, including asperspin A (**1**) and asperther A (**2**) (isolated from rice medium), together with seven known compounds, diorcinol-3-*O*-*α*-d-ribofuranoside (**3**) [15], 4-carbglyceryl-3,3’-dihydroxy-5,5’-dimethyldiphenyl ether (**4**) [16], gibellulin B (**5**) [17], daldinin C (**6**) [18], 12-hydroxysydonic acid (**7**) [19], (+)-sydonic acid (**8**) [20], and oxalicumone A (**9**) [21] (Figure 1). Compounds **3**–**5** were detected in rice medium, while **6**–**9** were isolated from PDB medium. Herein we reported the isolation, structure elucidation and biological activities of these compounds.

## 2. Results and Discussion

### 2.1. Structure Elucidation

Asperspin A (**1**) was obtained as a white powder. Its molecular formula was determined as C_16_H_22_O_4_ by the negative HRESIMS ion at *m/z* 277.1440 [M − H]^−^ (calcd. for C_16_H_21_O_4_, 277.1445), corresponds to 6 degrees of unsaturation. The IR spectrum showed absorption bands for hydroxy (3390 cm^−1^) and aromatic ring (1645 cm^−1^) functionalities. The ^1^H NMR spectrum (Table 1) of **1** displayed the presence of a 1,2,3,4-tetrasubstituted benzene ring [*δ*_H_ 7.05 (1H, d, *J* = 7.7 Hz, H-4), 6.98 (1H, d, *J* = 7.7 Hz, H-5)]. The ^1^H NMR spectrum of **1** also showed a methoxy [*δ*_H_ 3.39 (s)], two methyls [*δ*_H_ 1.20 (s), 1.30 (s)], three methylenes [*δ*_H_ 4.75 (m), 4.09 (dd, *J* = 6.0, 1.6 Hz), 3.15 (m)], and three methines [*δ*_H_ 6.90 (d, *J* = 15.7 Hz), 6.16 (dt, *J* = 15.7, 5.9 Hz), and 4.61 (t, *J* = 9.0 Hz)]. The ^13^C NMR data (Table 1) of **1** contained 16 carbons comprising of six aromatic carbons, three methylenes, three methines, two methyls, a methoxy group and a quaternary carbon. Among them, a benzene ring and two olefinic carbons could be easily identified from the ^13^C NMR, which accounted for five degrees of unsaturation. The remaining one degree of unsaturation was due to the presence of a ring in the structure. The HMBC correlations from H-3 to C-3a and C-8b, from H-2 to C-8b and C-13 and the COSY correlation of H-2/H-3 indicated the existence of a furan ring fused with a benzene ring through C-3a-C-8b and placed a quaternary carbon at C-13 (*δ*_C_ 72.6) at C-2. Two methyls at C-14 and C-15 were assigned at C-13, by the HMBC correlations of H-14 and H-15/C-13, respectively. The substituted hydroxymethylene group C-8 was attached to C-8a, according to the HMBC correlations of H-8/8a and 8b. Moreover, the COSY correlations of H-6/H-9 and H-9/H-10 and the HMBC correlations from H-6 to C-10 and from H-10 to C-12 indicated that **1** possessed a methoxypropylene fragment. Furthermore, the methoxypropylene moiety was positioned at C-5a based on the HMBC correlation of H-9/C-5a. The configuration of the double bond between C-6 and C-9 was assigned as *E* form according to a NOESY correlation between H-6 and H-10, as well as a large coupling constant of 15.7 Hz. The absolute configuration of the C-2 chiral center in **1** was determined to be *R* by comparing the optical rotation value ([α${]}_{D}^{25}$ −4.0) with that of alcohol [(−)−V] previously reported in the literature [22]. Detailed data can be found in Figures S3–S9.

**Figure 2:**
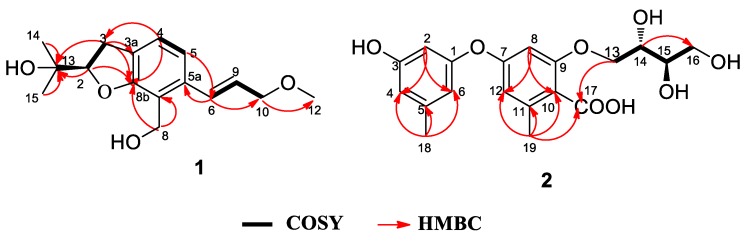
Key HMBC, COSY correlations of **1** and **2**.

Asperther A (**2**) was isolated as a yellow oil. The molecular formula was established as C_19_H_22_O_8_ on the basis of the negative HRESIMS ion at m/z 377.1237 [M − H]^−^ (calcd. for C_19_H_2__1_O_8_, 377.1242), indicating 9 indices of hydrogen deficiency. The IR spectrum showed bands due to hydroxy (3356 cm^−1^) and aromatic ring (1598 cm^−1^) functions. The ^1^H NMR data of **2** (Table 2) displayed the presence of five aromatic [*δ*_H_ 6.29 (t, *J* = 2.2 Hz), 6.49 (t, *J* = 1.6 Hz), 6.36 (d, *J* = 2.5 Hz), 6.24 (d, *J* = 2.5 Hz), 6.37 (s)], and six aliphatic protons [*δ*_H_ 4.42 (dd, *J* = 11.6, 6.6 Hz), 4.62 (dd, *J* = 11.6, 2.8 Hz), 3.91 (td, *J* = 2.7, 6.7 Hz), 3.65 (td, *J* = 2.8, 6.9 Hz), 3.80 (dd, *J* = 13.8, 11.4 Hz), 3.67 (dd, *J* = 13.8, 5.4 Hz)], and two methyls [*δ*_H_ 2.27 (s), 2.52 (d, *J* = 7.7 Hz)]. The ^13^C NMR data (Table 2) displayed 19 carbon resonances, comprising 12 aromatic carbons, a carbonyl (*δ*_C_ 172.4), two methyls (*δ*_C_ 21.4, 24.5), two methylenes (*δ*_C_ 64.5, 68.1) and two methines (*δ*_C_ 71.0, 73.7). A meta-location of protons H-2, H-4, H-6, H-8 and H-12 were confirmed by the coupling constants of H-2 [*δ*_H_ 6.29 (1H, t, J = 2.2 Hz)], H-8 [*δ*_H_ 6.24 (1H, d, *J* = 2.5 Hz)], and H-12 [*δ*_H_ 6.36 (1H, d, *J* = 2.5 Hz)]. The mutual HMBC correlations between CH-2, CH-4 and CH-6, and HMBC correlations from H-2 to C-1 and C-3, from H-4 to C-3, C-5 and C-18, from H-6 to C-1, C-5, and C-18, and from H-18 to C-4 and C-6 indicated the existence of an orcinolic unit in **2**. The *ortho*-orselinic acid moiety in 2 was determined based on the HMBC correlations of H-8/C-7, C-9, C-10, and C-12, of H-12/C-7, C-8, C-10 and C-19, of H-19/C-10, C-11, C-12 and C-17 (Figure 2). The COSY correlations of H-13/H-14; H-14/H-15; H-15/H-16 revealed a butane-1,2,3,4-tetrol moiety in 2. Moreover, the butane-1,2,3,4-tetrol moiety was connected to *ortho*-orselinic moiety through C-13–O–C-9 by the HMBC correlation of H-13/C-17. The butane-1,2,3,4-tetrol moiety was assigned to be erythritol by comparison of H-2 and H-3 coupling constants (*J* = 6.8 Hz) with the rule reported by Hawkes [23]. Furthermore, the absolute configuration of **2** was established as 2*S*,3*R* by comparing the optical rotation of 2 (−7.4) with that of (2*S*,3*R*)-montagnetol (−10.1) [24,25].

Furthermore, seven known compounds **3**–**9** were identified as diorcinol-3-*O*-*α*-D-ribofuranoside (**3**) [15], 4-carbglyceryl-3,3’-dihydroxy-5,5’-dimethyldiphenyl ether (**4**) [16], gibellulin B (**5**) [17], daldinin C (**6**) [18], 12-hydroxysydonic acid (**7**) [19], (+)-sydonic acid (**8**) [20], and oxalicumone A (**9**) [21] by comparison of their spectral data with those reported in the literature.

### 2.2. Biological Activities

The antibacterial activity was tested using sequential 2-fold serial dilutions of each compound in DMSO to provide 10 concentrations for all of the assays [26]. Four pathogenic bacteria, including *V. parahaemolyticus, V. harveyi, E. coli,* and *S. aureus*, were used, and the chloramphenicol was used as positive control. The results (Table 3) revealed that **9** showed weak inhibitory activity against *E. coli* with an MIC value of 75.4 µM. **7** had significant inhibitory activity against *S. aureus* (MIC value, 3.54 µM). Other compounds had no significant activity against four pathogenic bacteria.

Compounds **1**–**9** were also evaluated for their cytotoxic activities against seven human-derived cancer cell lines CCRF-CEM (human acute lymphoblastic leukemia T lymphocyte), K562 (human chronic myeloid leukemia cell), BGC823 (human gastric adenocarcinoma), AGS (human gastric adenocarcinoma),HCT-116 (human colon cancer cell), MDA-MB-453 (human breast cancer cell), and COR-L23 (human lung cancer cell) using the 3-(4, 5-dimethylthiazol-2-yl)-2, 5-diphenyltetrazolium bromide (MTT) assay and following the procedure previously described [27]. None of the compounds showed cytotoxicity against the BGC823 and AGS. Compound **1**, **2**, **5**, and **6** exhibited weak effects against human cancer cell lines HCT-116 (**1**), CCRF-CEM (**2**), K562 (**2**), MDA-MB-453 (**5**), and COR-L23 (**6**). Compound **9** exhibited pronounced cytotoxicity against CCRF-CEM cell and K562 cell with the IC_50_ values of 1.22 ± 0.045 and 9.58 ± 0.19 µM, respectively. The results (Table 4) revealed that these compounds were found to display highly selective antibacterial activities and cytotoxicities.

## 3. Materials and Methods

### 3.1. General Experimental Procedures

Optical rotations were measured with a P-2000 digital polarimeter (JASCO, Hachioji, Japan). UV spectra were obtained with a NADE Evolution 201 spectrophotometer (ThermoFisher, Waltham, MA, USA). IR spectra were recorded on a Nicolet iS5 IR spectrometer (ThermoFisher, Waltham, MA, USA).^1^H-NMR (600 MHz) and ^13^C (150 MHz) spectra were measured with a Varian 600 MHz (Palo Alto, CA, USA) spectrometer. Chemical shifts were reported in ppm, while using the signals of the residual solvent as internal reference (*δ*_H_ 3.31 and *δ*_C_ 49.0 for CD_3_OD, *δ*_H_ 7.26 and *δ*_C_ 77.1 for CDCl_3_). HRESIMS data were recorded on an Agilent Technologies 6520 Accurate Mass Q-TOF LC/MS spectrometer (Agilent Technologies, Santa Clara, CA, USA). Medium-pressure liquid chromatography (MPLC) was performed on a FLEXA Purification System (Agela Technologies, Tianjin, China) using a ODS column. Column chromatography (CC) was carried out with silica gel (200–300 mesh, Qingdao Marine Chemical Inc. Qingdao, PR China) and Sephadex LH20 (Amersham Biosciences, Piscataway, NJ, USA). CC fractions were analyzed by TLC (silica gel GF254, Qingdao Marine Chemical Factory, Qingdao, China). Semi-preparative HPLC (Waters 600, Milford, MA, USA) equipped with a Waters 2996 detector and a C_18_ column (250 mm × 20 mm ID, 5 µm; YMC Co. Ltd., Tokyo, Japan).

### 3.2. Fungal Material

The fungal strain, *Aspergillus* sp. LS34, was isolated from the sponge *Haliclona* sp. collected at Lingshui, Hainan Province, China. It was identified as *Aspergillus* sp. according to morphological and molecular (ITS rDNA sequence) analyses (GenBank accession ID: EU645721, 99% similarity). A voucher specimen (No. LS34) was deposited in the potato dextrose agar (PDA) medium (potato 200 g, dextrose 20 g/L, sea salt 35 g/L and agarose 20 g/L) at Ningbo University, Ningbo, China.

### 3.3. Fermentation

After being maintained on PDA medium for 7 days, the strain LS34 was inoculated into 1 L Erlenmeyer flasks that contained 200 mL of PDB medium (potato 200 g, dextrose 20 g/L and sea salt 35 g/L) at 28 °C for 2 days on a rotary shake at 200 rpm. Then, the seed culture was inoculated into two media including potato dextrose broth (PDB) medium containing (potato 200 g, dextrose 20 g/L and sea salt 35 g/L, 30 flasks) on a rotatory shaker (180 rpm) at 28 °C for 12 days, and solid rice medium (rice 120 g and sea water 180 mL, 30 flasks) at 28 °C for 30 days under static conditions.

### 3.4. Extraction and Isolation

The PDB fermentation broth was extracted using EtOAc to afford crude extract (4.5 g), which was subjected to gel filtration on a Sephadex LH-20 column, eluted with CH_3_OH and CH_2_Cl_2_ (1:1, *v/v*), affording three fractions (Fr. 1–3). Fr. 2 (1.9 g) was subjected to vacuum liquid chromatography (VLC) on a silica gel column (6 × 15 cm, 200–300 mesh) eluting with mixtures of solvents with increasing polarity: hexane/EtOAc (from 20:1 to 0:1 (*v/v*)) to yield four subfractions (Fr.2.A–D), then EtOAc/MeOH (from 1:1 to 0:1 (*v/v*)) to yield two fractions (Fr.2.E–F). Fr.2.D (130 mg) was further separated by reversed-phase MPLC (30–100% MeOH/H_2_O, 120 min, flow rate 20 mL/min) to get four subfractions (Fr.2.D.1–4). Furthermore, subtraction Fr.2.D.2 was purified by semipreparative RP-HPLC (2.0 mL/min; 35% MeCN in H_2_O) to yield **6** (*t*_R_ 20.2 min, 3.6 mg) and **9** (*t*_R_ 32.4 min, 4.2 mg). Subfraction Fr.2.D.3 was further separated by semipreparative RP-HPLC (2.0 mL/min; 40% MeCN in H_2_O) to obtain **7** (*t*_R_ 25.8 min, 2.7 mg) and **8** (*t*_R_ 34.7 min, 4.9 mg).

The rice fermentation was exhaustively extracted with MeOH three times in an ultrasonic bath at 40 °C for 15 min and filtered, then MeOH layers were combined and evaporated. The extract was suspended in 1L H_2_O and extracted three times with EtOAc, after which it was filtered and evaporated *in vacuo* to obtain crude extracts (5.3 g). The crude extract was further separated by CC on Sephadex LH-20 eluting with CH_3_OH and CH_2_Cl_2_ (1:1, *v/v*) to afford three fractions (Fr. A–C). Fr. B was subjected to VLC over silica gel using gradients of hexane/EtOAc (from 20:1 to 0:1 (*v/v*)) to yield four subfractions (Fr.B.1–4), then EtOAc/MeOH (from 1:1 to 0:1 (*v/v*)) to yield two fractions (Fr.B.5–6). Fr.B.4 was further separated by reversed-phase MPLC (30–100% MeOH/H_2_O, 120 min, flow rate 20 mL/min) to afford three fractions (Fr.B.4.A–C). Furthermore, using semi-preparative RP-HPLC and elution with 30% MeCN, compounds **3** (*t*_R_ 27.5 min, 3.2 mg) and **4** (*t*_R_ 36.9 min, 7.7 mg) were isolated from Fr.B.4.B. Fr.B.5 was further separated by MPLC (30–100% MeOH/H_2_O, 120 min, flow rate 20 mL/min) to obtain five fractions (Fr.B.5.A–E). Subsequently, the subfraction Fr.B.5.B was purified by semipreparative RP-HPLC (2.0 mL/min; 30% MeCN in H_2_O) to yield **2** (*t*_R_ 20.2 min, 2.2 mg) and **5** (*t*_R_ 24.1 min, 6.7 mg). Subfraction Fr.B.5.C was purified by semipreparative RP-HPLC (2.0 mL/min; 40% MeCN in H_2_O) to afford **1** (*t*_R_ 27.3 min, 3.5 mg).

Asperspin A: White powder; molecular formula C_16_H_22_O_4_; [α${]}_{D}^{25}$ −4.0 (*c* 0.1, MeOH); UV (MeOH) *λ*_max_ (log ε) 220 (4.04), 305 (3.35) nm; IR (KBr) *ν*_max_ 3390, 2919, 1645, 1437, 968 cm^−1^; ^13^C and ^1^H NMR data (in CDCl_3_), see Table 1, HRESIMS *m/z* 277.1440 [M − H]^−^ (calcd. for C_16_H_21_O_4_, 277.1445).

Asperther A: Yellow oil; molecular formula C_19_H_22_O_8_; [α${]}_{D}^{25}$ −7.4 (*c* 0.1, MeOH); UV (MeOH) *λ*_max_ (log ε) 204 (4.21), 218 (4.65) nm; IR (KBr) *ν*_max_ 3356, 1647, 1598, 1459, 1318, 1258, 1205, 1161, 1029 cm^−1^; ^13^C and ^1^H NMR data (in CD_3_OD), see Table 2, HRESIMS *m/z* 377.1237 [M − H] ^−^ (calcd. for C_19_H_21_O_8_, 377.1242).

### 3.5. Biological Assay

The antibacterial assay was conducted using the conventional broth dilution method [26]. Briefly, a series of different concentrations of the test compounds were dissolved to DMSO, using sequential 2-fold serial dilutions to obtain different concentrations. Each concentration of test compounds was added to 96-well plates, then bacterial suspension (10^6^ CFU per milliliter) was added to the plate and was kept at 28 °C for 48 h. All the procedures in the assay were performed in triplicates. The minimum inhibitory concentration (MIC) for *V parahaemolyticus*, *V harveyi*, *E. coli, and S. aureus* were determined according to the concentration that inhibited visible growth of pathogen. The cytotoxic activities of compounds **1**–**9** against human cancer cell lines were assessed by the MTT method, as described previously [27]. In brief, the cell suspension (1 × 10^5^/mL) was inoculated into 96-well plates and was kept at 37 °C for 12 h. Then each well was added with sample solvent and further cultured at 37 °C for 48 h. Subsequently, MTT was added to each well and incubated at 37 °C for 4 h. After removing the medium, the cells were lysed with 20% SDS-50% DMF. Absorbance of each well was measured using a 96-well microplate reader at 595 nm for assessment of cell growth. Chidamide was used as a positive control.

## 4. Conclusions

Based on the OSMAC culture strategy, the chemical investigation of marine-derived fungus *Aspergillus* sp. LS34 resulted in isolation of isolation of two new compounds **1** and **2** together with three known compounds **3**–**5** from solid rice medium, and four known compounds **6**–**9** from the PDB medium. Obviously, the OSMAC strategy represented a powerful way to induce new metabolites from microorganisms. Diphenyl ethers **2**–**5** and their derivatives have been extensively investigated and exhibited variability due to the diversity and location of hydroxyl group and the side chain. Compound **3** was a rare diphenyl ether derivative containing a d-ribofuranose fragment. Notably, compound **7** displayed pronounced antibacterial activity against *S. aureus*. Compound **9** exhibited significant cytotoxic activity against human cancer cell lines CCRF-CEM and K562.

## Figures and Tables

**Table 1:** ^1^H and ^13^C NMR data of compound **1** (600 MHz, 150 MHz, in CDCl_3_).

Position	*δ*_C_	*δ*_H_ (*J* in Hz)
2	89.8	4.61 (1H, t, 9.0)
3	30.8	3.15 (2H, m)
3a	126.7	
4	124.5	7.05 (1H, d, 7.7)
5	119.2	6.98 (1H, d, 7.7)
5a	136.3	
6	129.4	6.90 (1H, d, 15.7)
8	56.8	4.75 (2H, m)
8a	119.5	
8b	158.6	
9	128.5	6.16 (1H, dt, 15.7, 5.9)
10	73.3	4.09 (2H, dd, 6.0, 1.6)
12	58.2	3.39 (3H, s)
13	72.6	
14	24.3	1.20 (3H, s)
15	26.4	1.30 (3H, s)

**Table 2:** ^1^H and ^13^C NMR data of compound **2** (600 MHz, 150 MHz, in CD_3_OD).

Position	*δ*_C_	*δ*_H_ (*J* in Hz)
1	157.4	
2	105.7	6.29 (1H, t, 2.2)
3	159.9	
4	113.6	6.49 (1H, t, 1.6)
5	142.1	
6	113.1	6.37 (1H, s)
7	163.5	
8	103.7	6.24 (1H, d, 2.5)
9	164.5	
10	109.5	
11	144.6	
12	113.4	6.36 (1H, d, 2.5)
13	68.1	4.42 (1H, dd, 11.6, 6.6) 4.62 (1H, dd, 11.6, 2.8)
14	71.0	3.91 (1H, td, 2.8, 6.7)
15	73.7	3.65 (1H, td, 2.8, 6.9)
16	64.5	3.80 (1H, dd, 13.8, 11.4) 3.67 (1H, dd, 13.8, 5.4)
17	172.4	
18	21.4	2.27 (3H, s)
19	24.5	2.52 (3H, d, 7.7)

**Table 3:** Antibacterial activities of compounds **1**, **2**, **5**, **7** and **9**.

Compounds	MIC (µM)			
	*V. parahaemolyticus*	*V. harveyi*	*E. coli*	*S. aureus*
**1**	/	/	230	460
**2**	/	/	/	170
**5**	/	/	/	492
**7**	453	/	/	3.54
**9**	/	301	75.4	/
Chloramphenicol	1.42	1.07	1.25	0.91

“/” no antibacterial activity.

**Table 4:** Cytotoxicities of compounds **1**, **2**, **5**, **6** and **9**.

Compound	IC_50_ (μM)				
	CCRF-CEM	K562	HCT-116	MDA-MB-453	COR-L23
**1**	/	/	27.63 ± 1.25	/	/
**2**	19.73 ± 0.98	29.28 ± 0.75	/	/	/
**5**	/	/	/	/	29.17 ± 0.98
**6**	/	/	/	22.58 ± 0.42	/
**9**	1.22 ± 0.05	10.58 ± 0.19	/	/	/
Chidamide	0.97 ± 0.1	1.87 ± 0.29			
5-Fluorouracil			2.7 ± 0.54	1.58 ± 0.42	6.62 ± 0.68

“/” no cytotoxicity.

## Supplementary Materials

The following are available online at https://www.mdpi.com/1660-3397/17/5/283/s1, Figures S1–S34: HRESIMS, HPLC chromatograms, 1D and 2D NMR, IR, or UV spectra of **1**–**9**.
