# Supplementary material for: Isolation and Characterization of Two New Metabolites from the Sponge-Derived Fungus Aspergillus sp. LS34 by OSMAC Approach
Source: Mar Drugs. 2019 May 11;17(5):283. doi: 10.3390/md17050283 (PMC6562850; doi:10.3390/md17050283)
Supplement: Supplementary file 1 [file marinedrugs-17-00283-s001.pdf]

marine drugs

Supplementary Material

Isolation and Characterization of Two New Metabolites from the Sponge-Derived Fungus *Aspergillus* sp. LS34 by OSMAC Approach

Wei Li, Lijian Ding*, Ning Wang, Jianzhou Xu, Weiyan Zhang, Bin Zhang, Shan He, Haixiao Jin*

Li Dak Sum Yip Yio Chin Kenneth Li Marine Biopharmaceutical Research Center, College of Food and Pharmaceutical Sciences, Ningbo University 315800, Ningbo, P. R. China; 15058882124@163.com (W.L.); wangning2@nbu.edu.cn (N.W.); 15669221063@163.com (J.X.); zhangbin1@nbu.edu.cn (B.Z); zhangweiyan@nbu.edu.cn (W.Z.); heshan@nbu.edu.cn (S.H.)

* Correspondence: dinglijian@nbu.edu.cn (L.D.); jinhaixiao@nbu.edu.cn (H.J.); Tel.: +86-574-8760-4388 (L.D.); +86-574-8760-4388 (H.J.)

The List of Contents

No.	Content	Page
1	Figure S1. IR spectrum of **1**	3
2	Figure S2. UV spectrum of compound **1**	3
3	Figure S3. ^{1}H NMR spectrum of compound **1** in $CDCl_3$	4
4	Figure S4. ^{13}C NMR spectrum of compound **1** in $CDCl_3$	4
5	Figure S5. DEPT 135 spectrum of compound **1** in $CDCl_3$	5
6	Figure S6. HSQC spectrum of compound **1** in $CDCl_3$	5
7	Figure S7. HMBC spectrum of compound **1** in $CDCl_3$	6
8	Figure S8. COSY spectrum of compound **1** in $CDCl_3$	6
9	Figure S9. HRESIMS of compound **1**	7
10	Figure S10. IR spectrum of compound **2**	7
11	Figure S11. UV spectrum of compound **2**	7
12	Figure S12. 1H NMR spectrum of compound **2** in CD_3OD	8
13	Figure S13. 13C NMR spectrum of compound **2** in CD_3OD	8
14	Figure S14. DEPT 135 spectrum of compound **2** in CD_3OD	9
15	Figure S15. HSQC spectrum of compound **2** in CD_3OD	9
16	Figure S16. HMBC spectrum of compound **2** in CD_3OD	10
17	Figure S17. COSY spectrum of compound **2** in CD_3OD	10
18	Figure S18. HRESIMS of compound **2**	11
19	Figure S19. ^{1}H NMR spectrum of compound **3** in CD_3OD	11
20	Figure S20. ^{13}C NMR spectrum of compound **3** in CD_3OD	12
21	Figure S21. ^{1}H NMR spectrum of compound **4** in DMSO-d_6	12
22	Figure S22. ^{13}C NMR spectrum of compound **4** in DMSO-d_6	13
23	Figure S23. ^{1}H NMR spectrum of compound **5** in CD_3OD	13
24	Figure S24. ^{13}C NMR spectrum of compound **5** in CD_3OD	14
25	Figure S25. ^{1}H NMR spectrum of compound **6** in CD_3OD	14
26	Figure S26. ^{13}C NMR spectrum of compound **6** in CD_3OD	15
27	Figure S27. ^{1}H NMR spectrum of compound **7** in CD_3OD	15
28	Figure S28. ^{13}C NMR spectrum of compound **7** in CD_3OD	16
29	Figure S29. ^{1}H NMR spectrum of compound **8** in $CDCl_3$	16
30	Figure S30. ^{13}C NMR spectrum of compound **8** in $CDCl_3$	17
31	Figure S31. ^{1}H NMR spectrum of compound **9** in $CDCl_3$	17
32	Figure S32. ^{13}C NMR spectrum of compound **9** in CDCl3	18
33	Figure S33. HPLC analysis chromatogram of compound **1**	18
34	Figure S34. HPLC analysis chromatogram of compound **2**	18

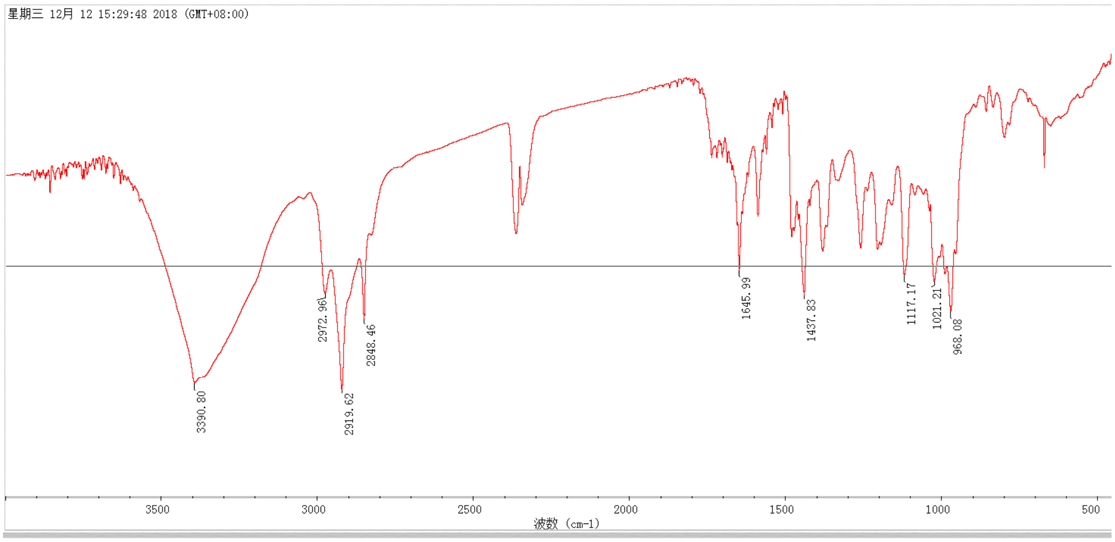

Figure S1. IR spectrum of compound **1**

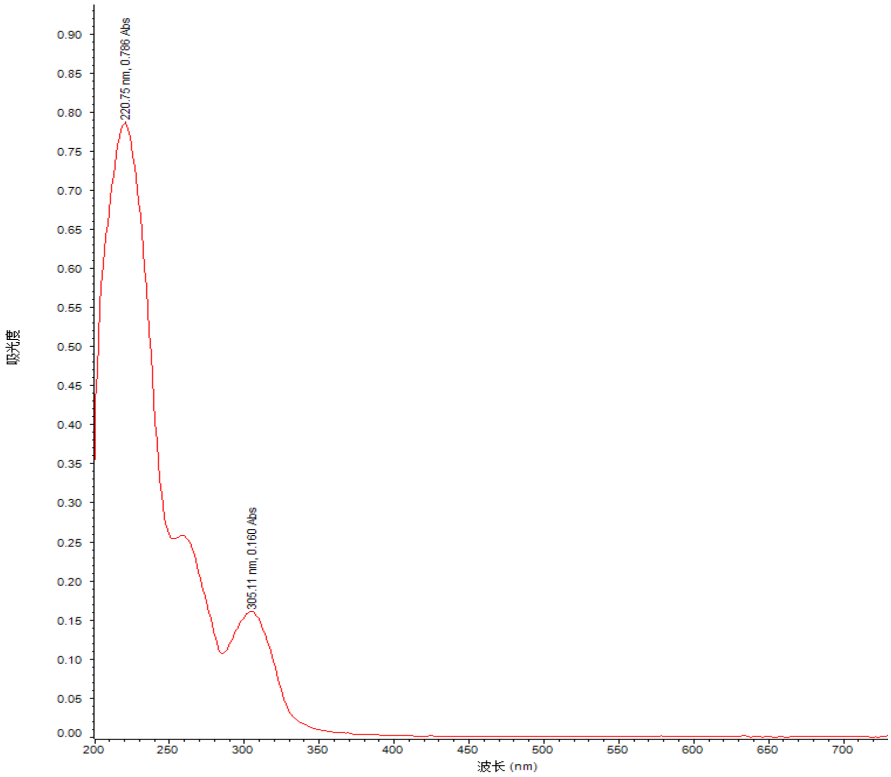

Figure S2. UV spectrum of compound **1**

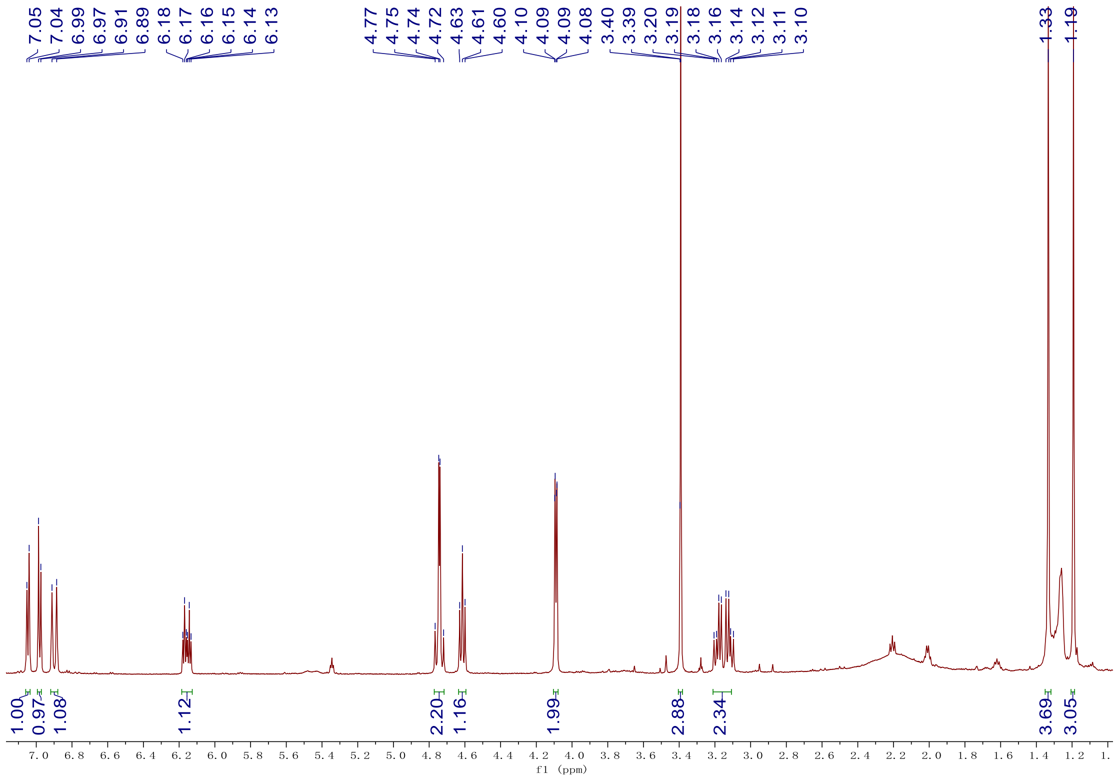

Figure S3. ^{1}H NMR spectrum of compound **1** in $CDCl_3$ (600 MHz)

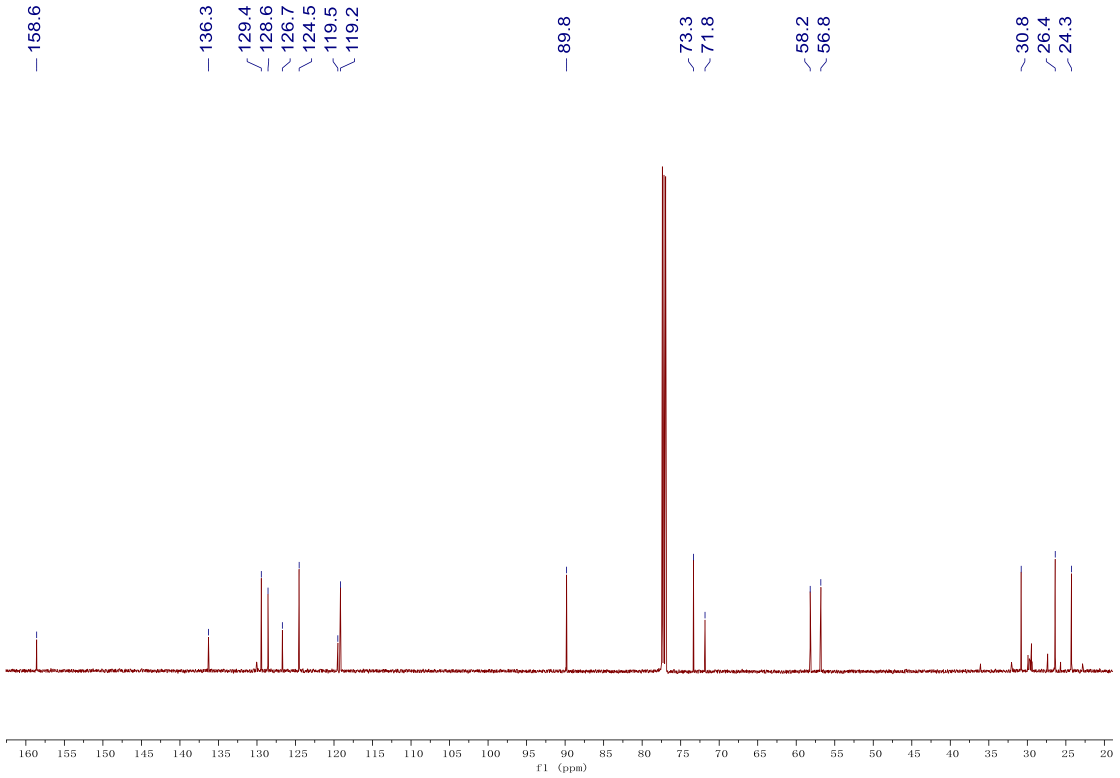

Figure S4. ^{13}C NMR spectrum of compound **1** in $CDCl_3$ (150 MHz)

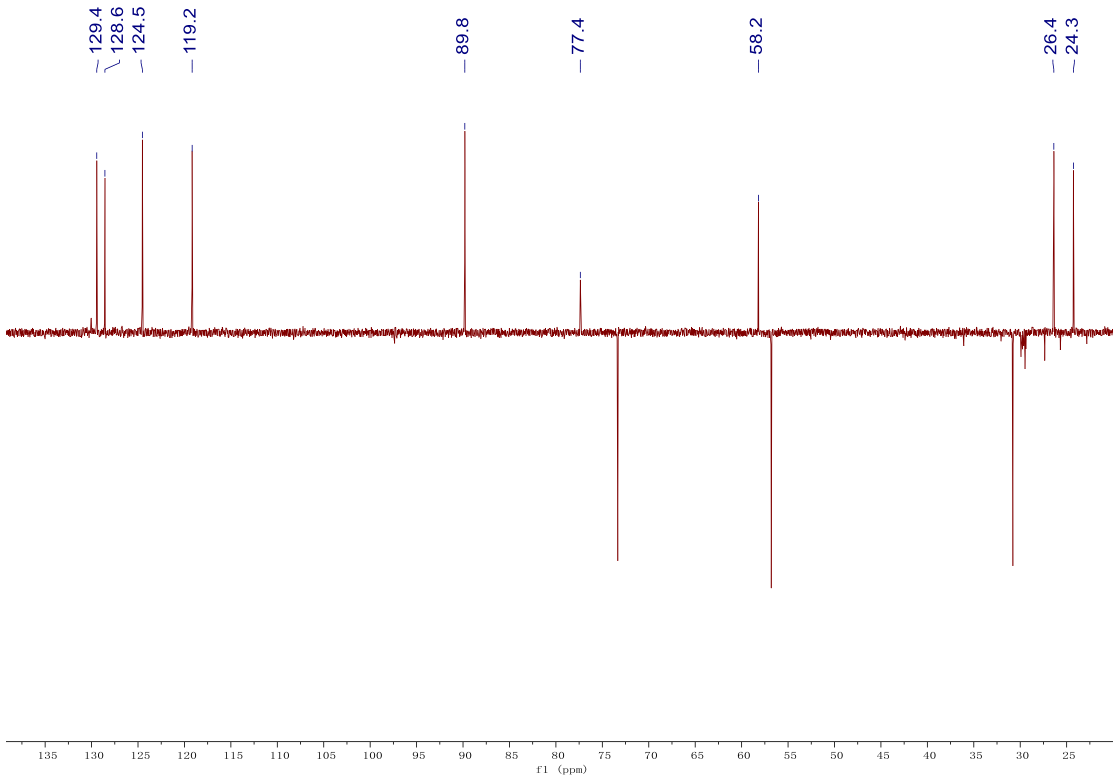

Figure S5. DEPT spectrum of compound 1 in $CDCl_3$

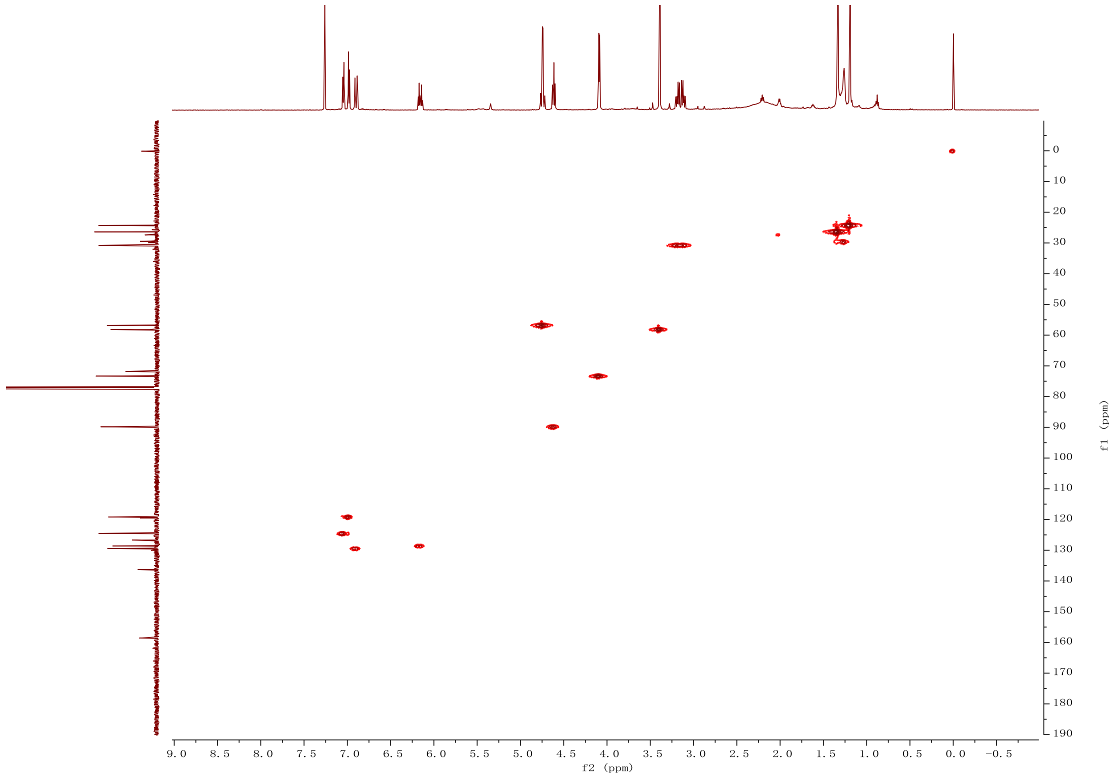

Figure S6. HSQC spectrum of compound **1** in $CDCl_3$

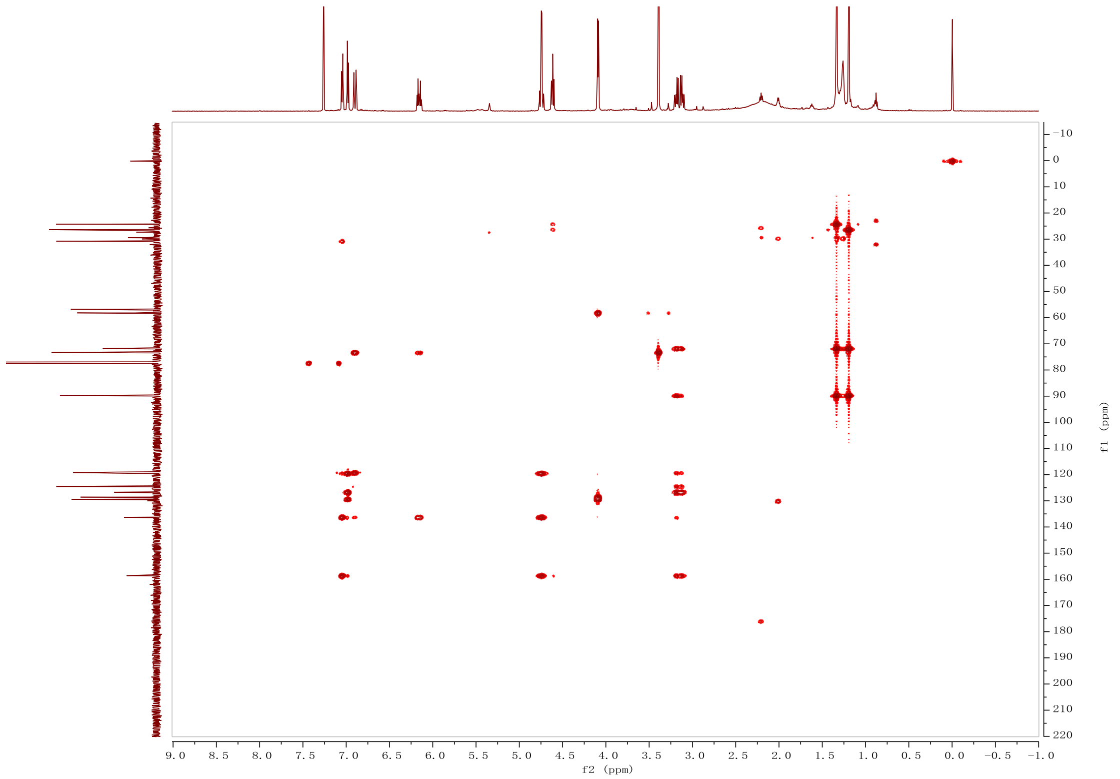

Figure S7. HMBC spectrum of compound **1** in $CDCl_3$

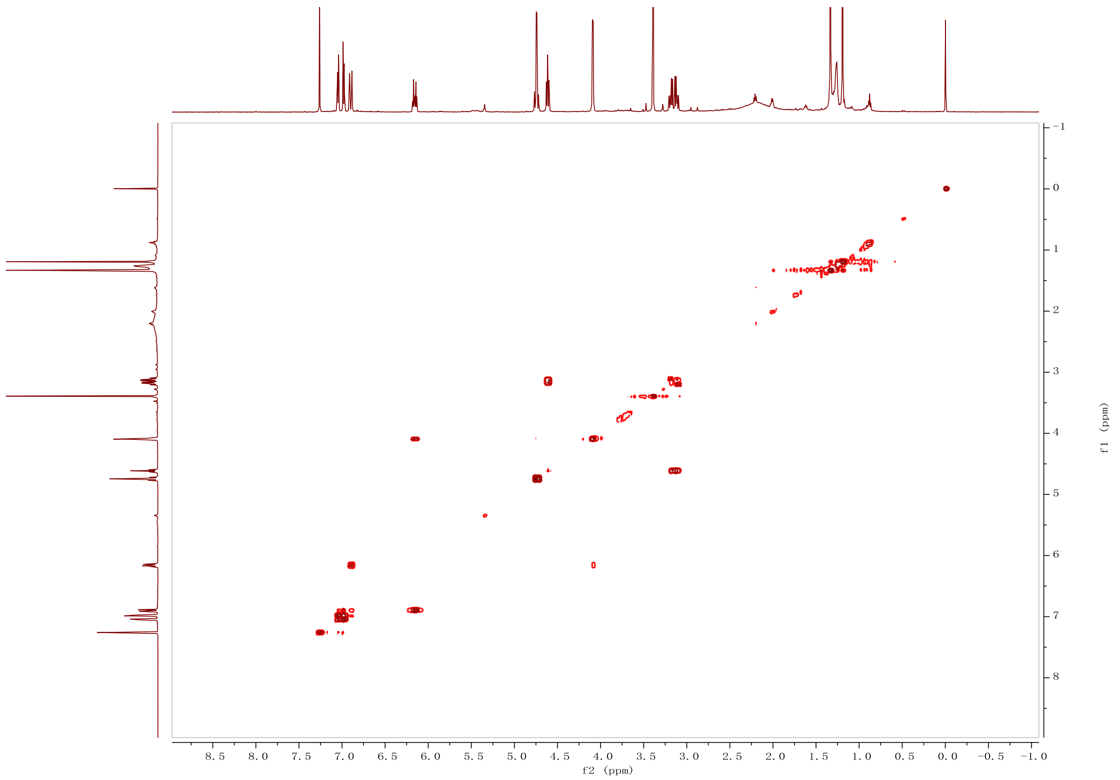

Figure S8. COSY spectrum of compound **1** in $CDCl_3$

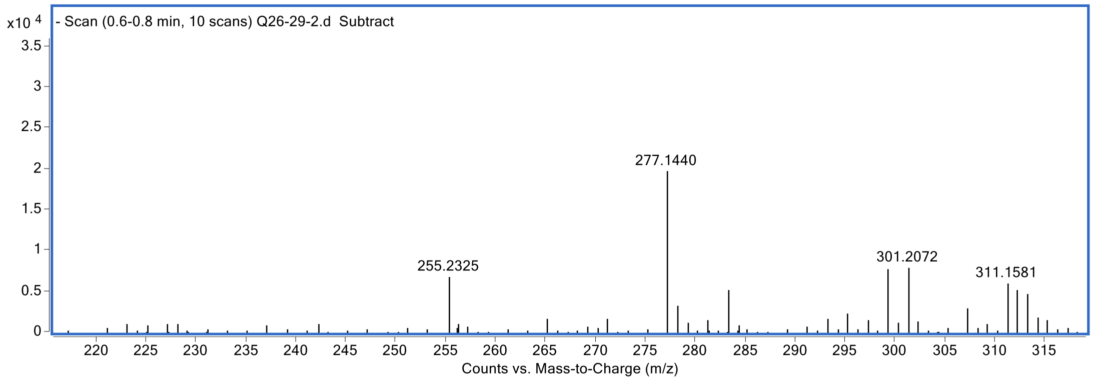

Formula (M)	Ion Formula	m/z	Calc m/z	Diff (ppm)	DBE
$C_{16}H_{22}O_4$	$C_{16}H_{21}O_4$	277.144	277.1445	1.92	6

Figure S9. HRESIMS of compound **1**

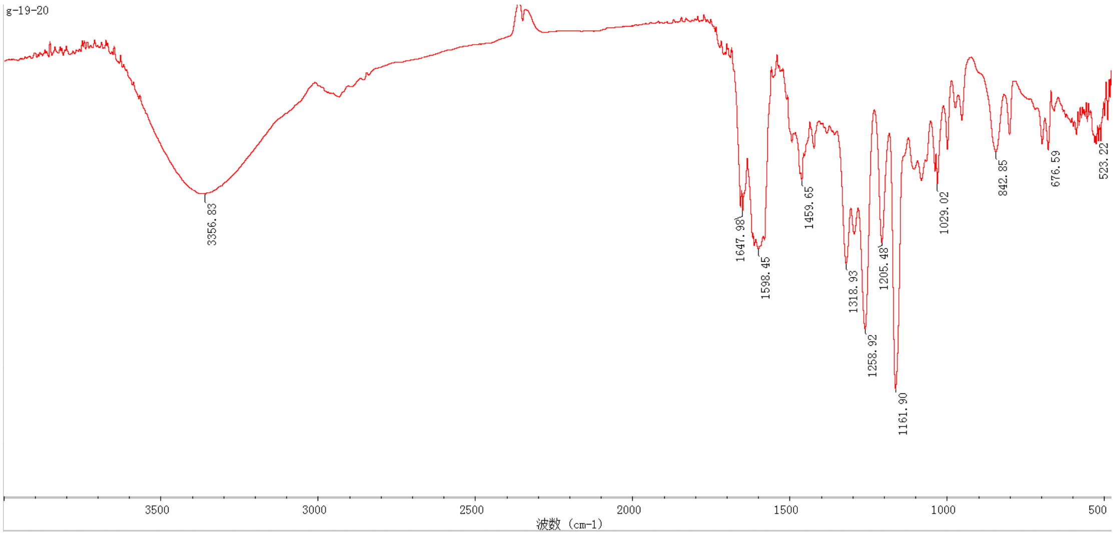

Figure S10. IR spectrum of compound **2**

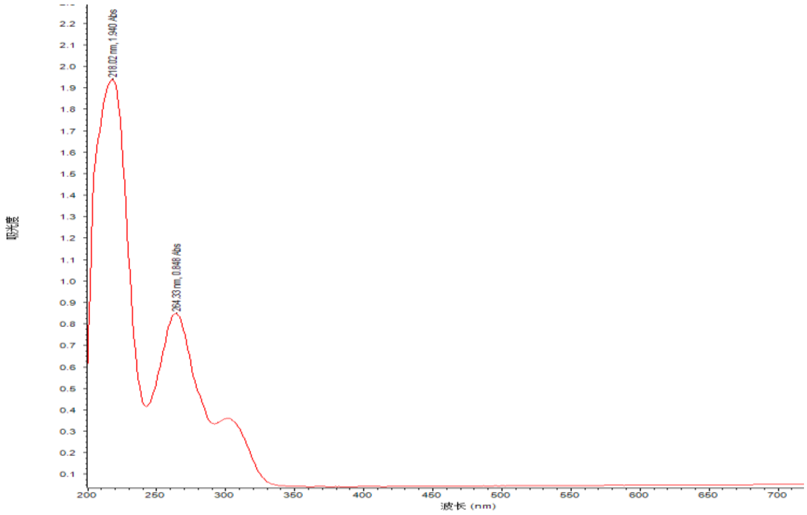

Figure S11. UV spectrum of compound **2**

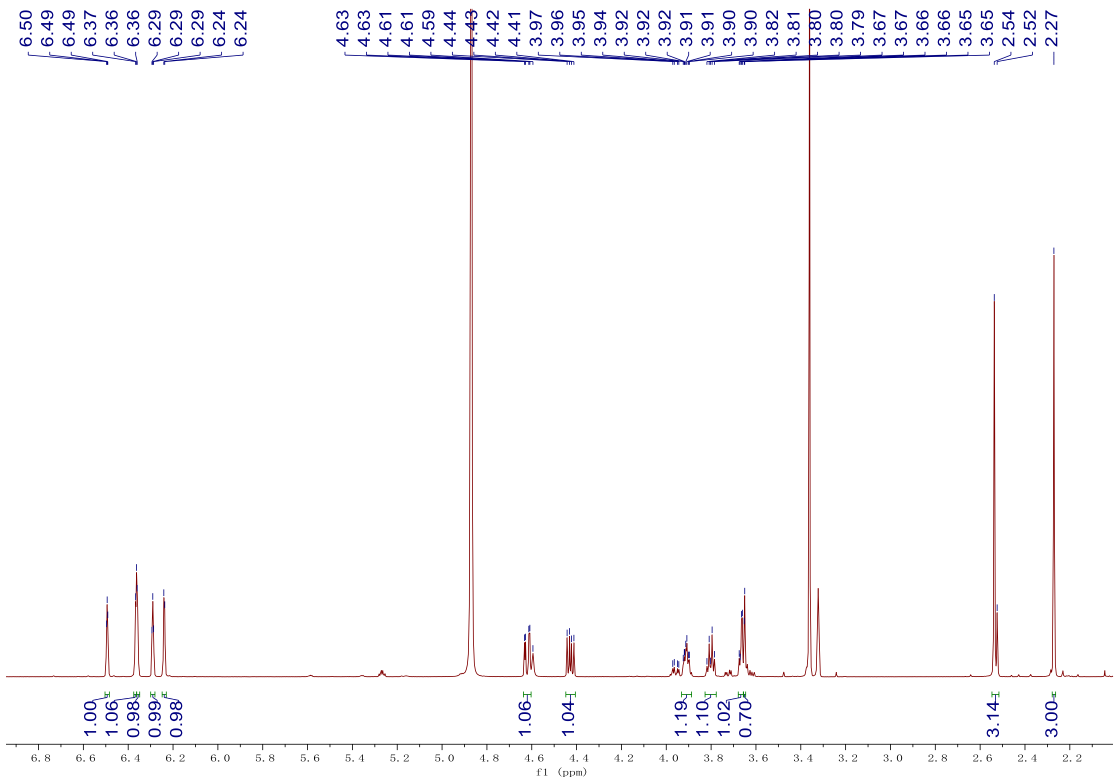

Figure S12. ^{1}H NMR spectrum of compound **2** in CD_3OD (600 MHz)

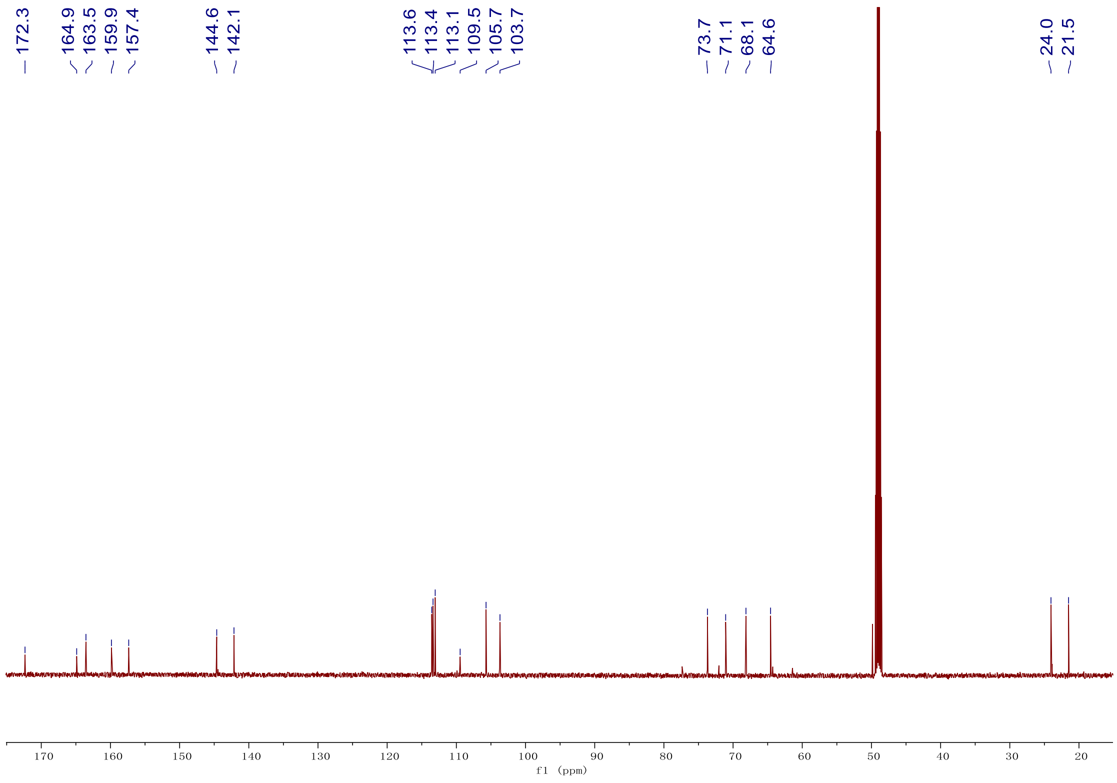

Figure S13. ^{13}C NMR spectrum of compound **2** in CD_3OD (150 MHz)

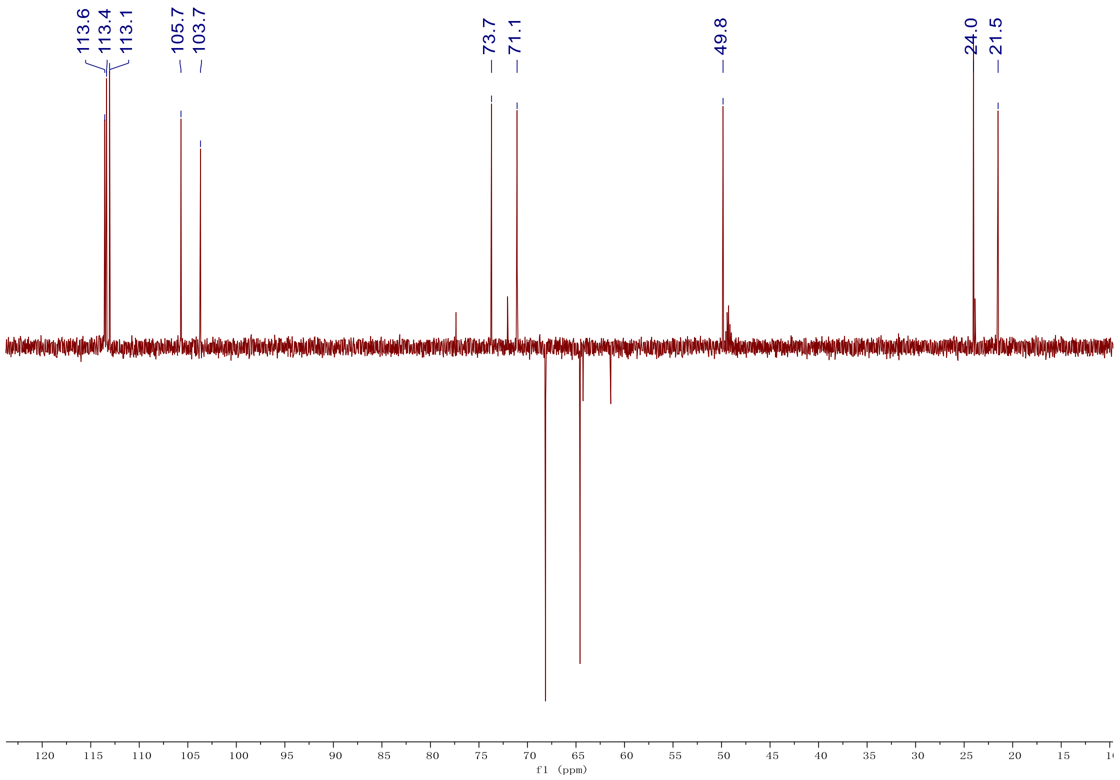

Figure S14. DEPT 135 spectrum of compound **2** in CD_3OD

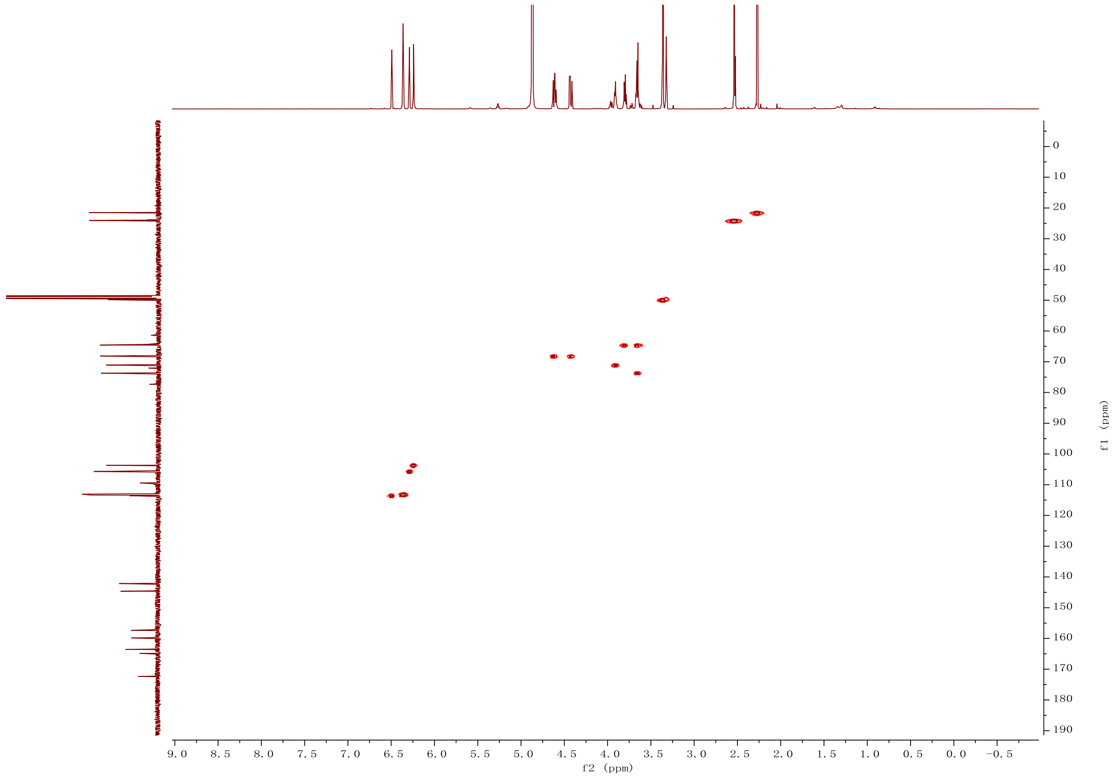

Figure S15. HSQC spectrum of compound **2** in CD_3OD

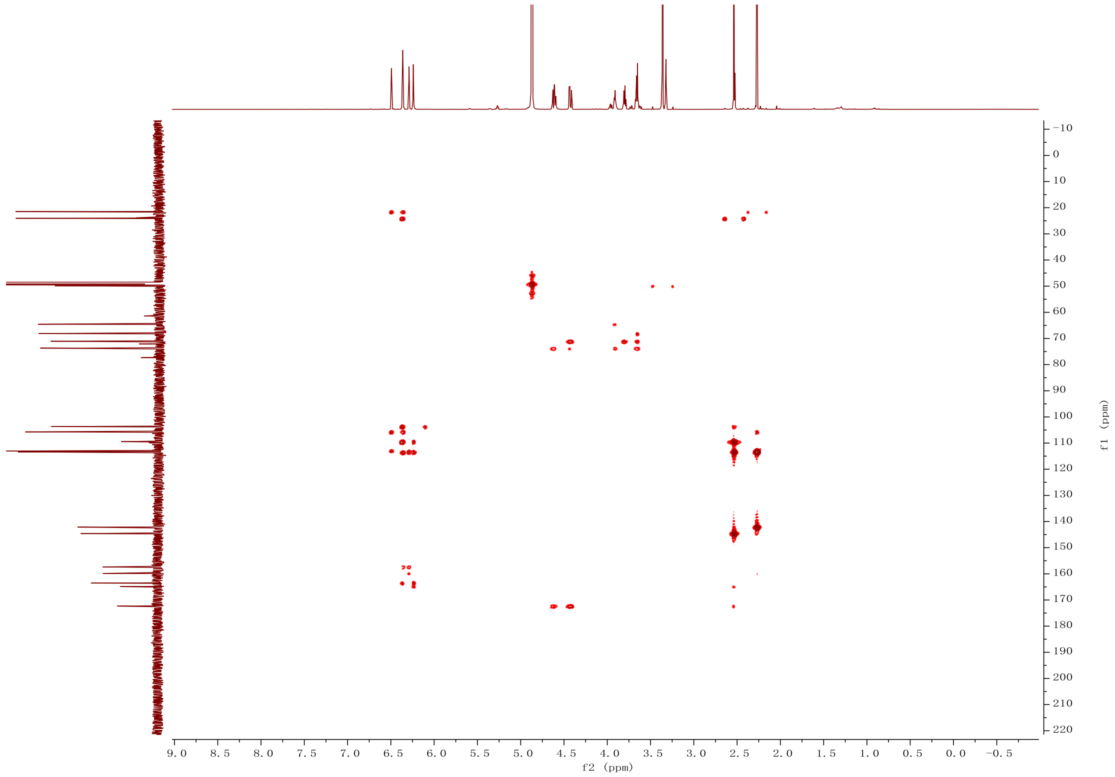

Figure S16. HMBC spectrum of compound **2** in CD_3OD

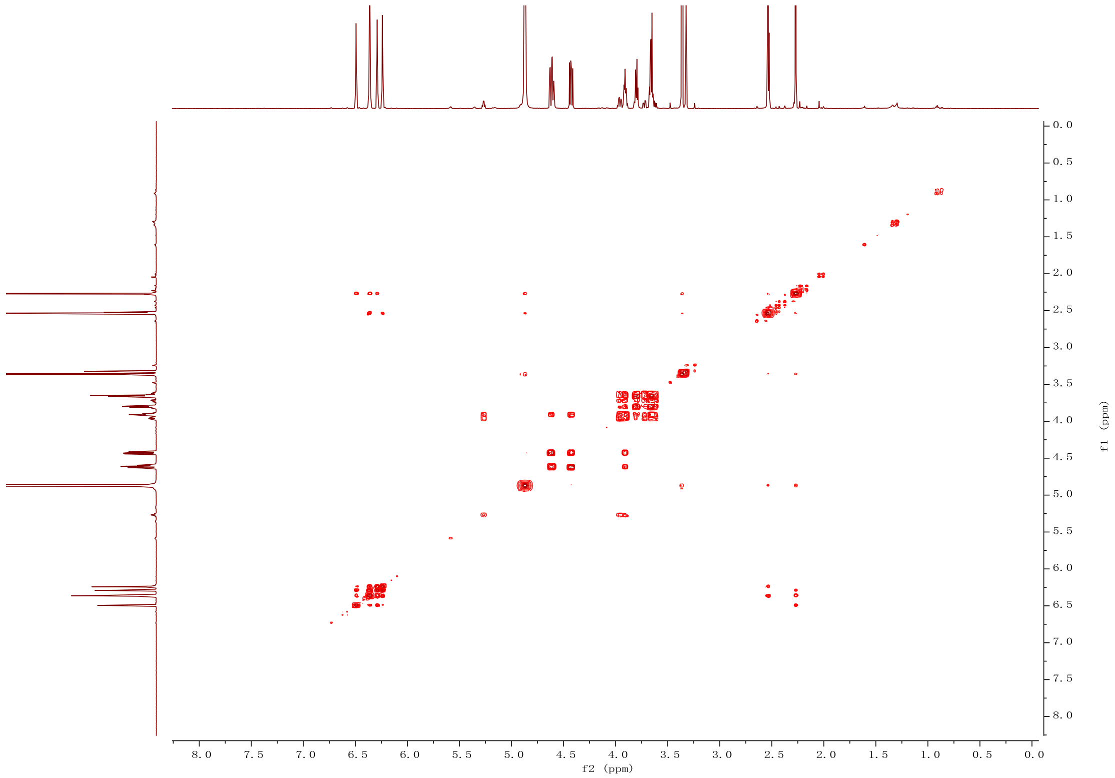

Figure S17. COSY spectrum of compound **2** in CD_3OD

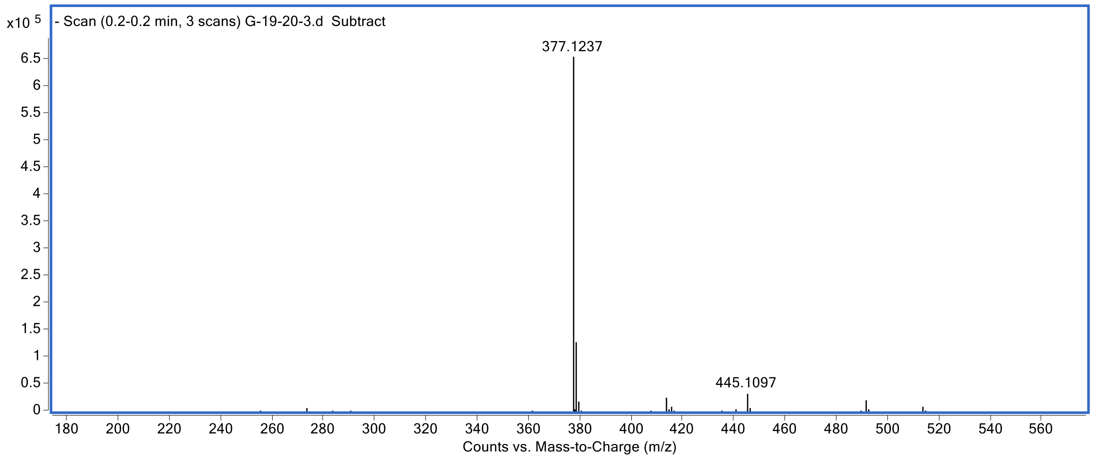

Formula (M)	Ion Formula	m/z	Calc m/z	Diff (ppm)	DBE
$C_{19}H_{22}O_8$	$C_{19}H_{21}O_8$	377.1237	377.1242	1.3	9

Figure S18. HRESIMS of compound **2**

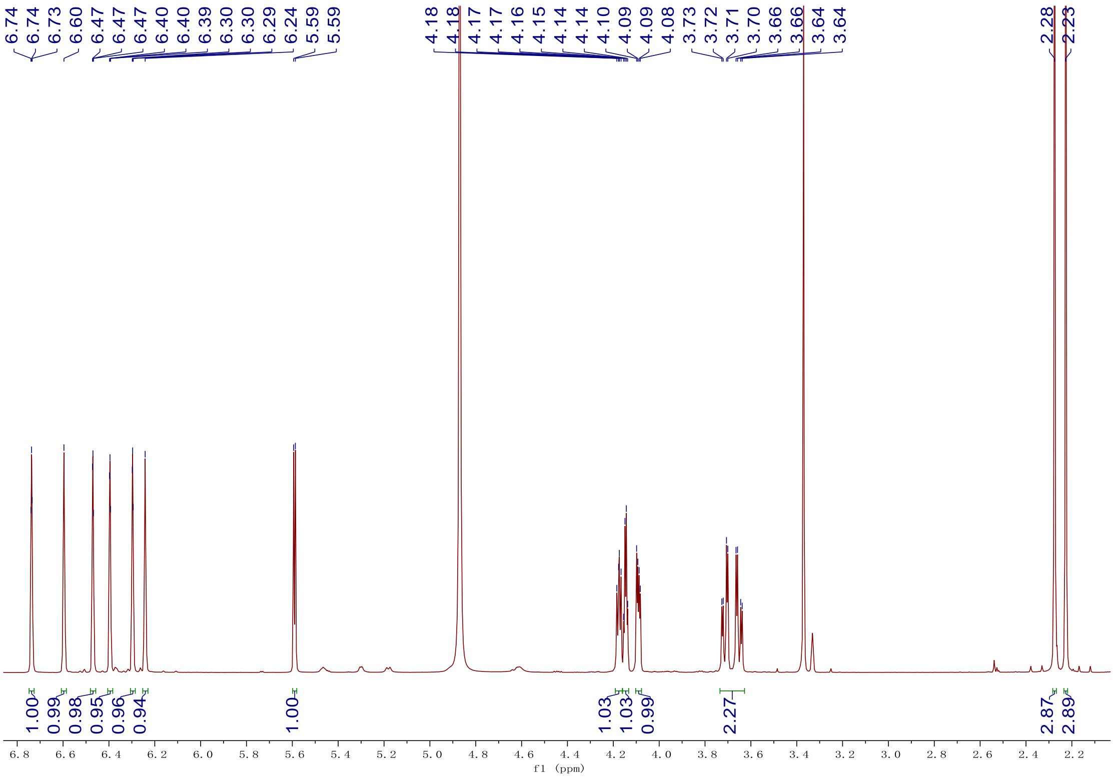

Figure S19. ^1H NMR spectrum of compound **3** in CD_3OD (600 MHz)

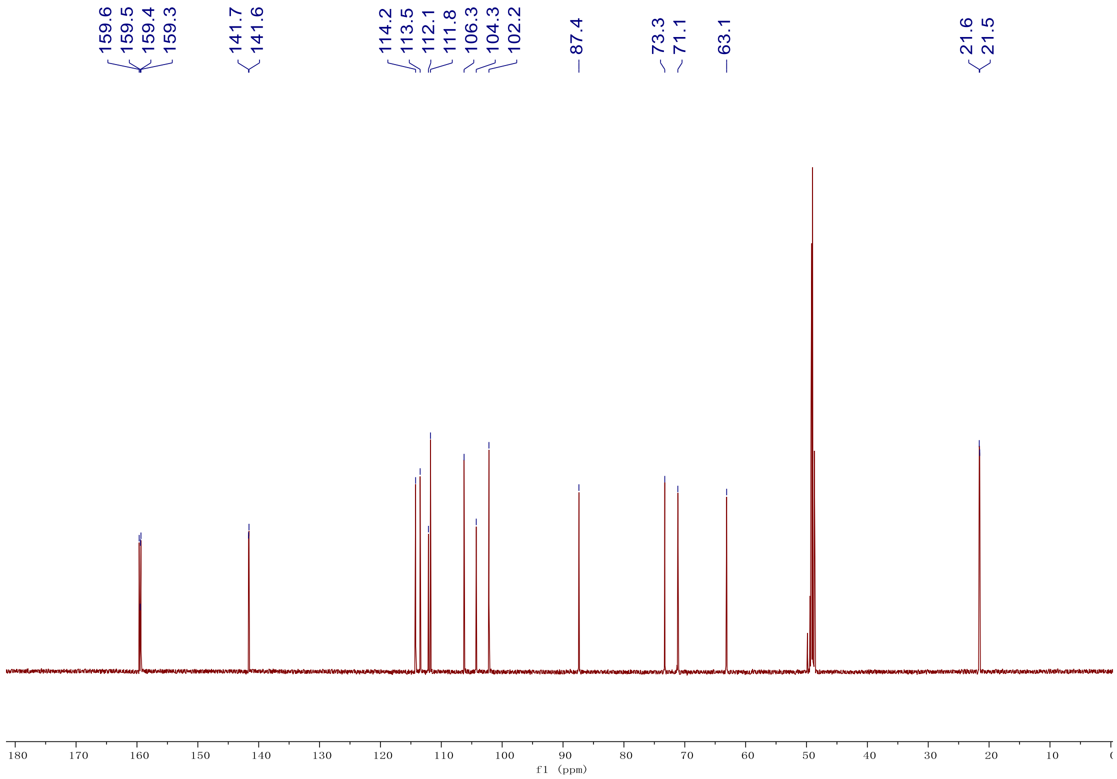

Figure S20. ^{13}C NMR spectrum of compound **3** in CD_3OD (600 MHz)

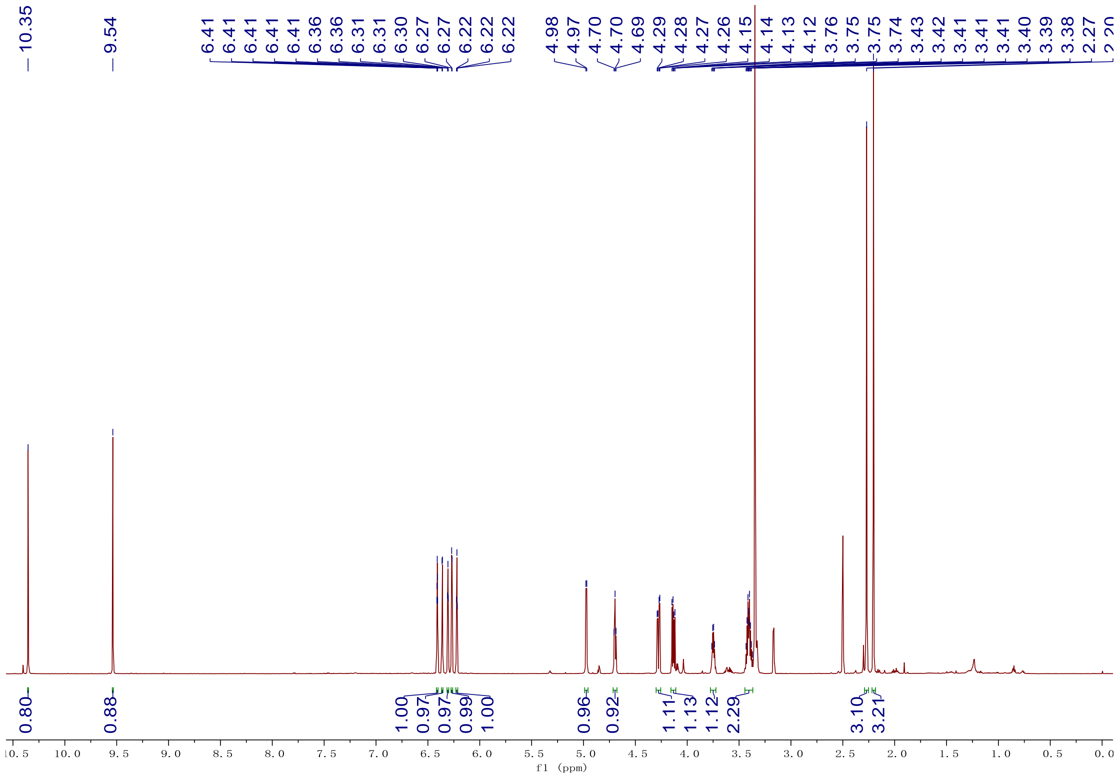

Figure S21. ^{1}H NMR spectrum of compound **4** in DMSO-d_6 (600 MHz)

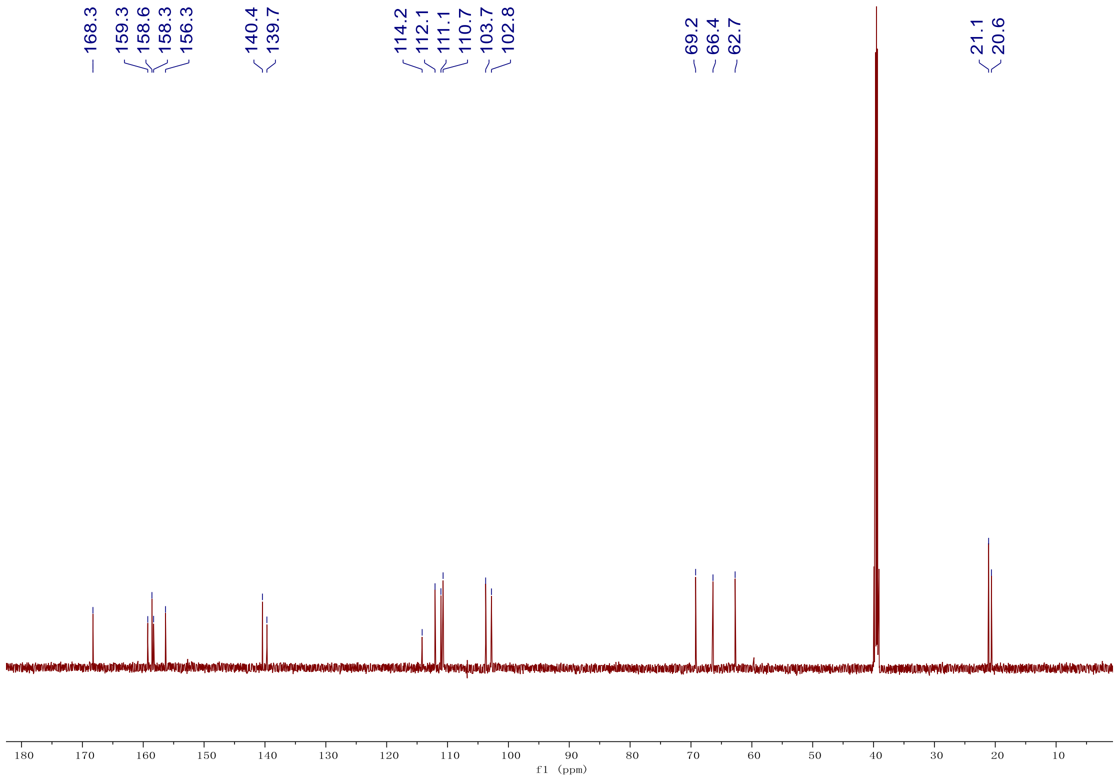

Figure S22. ^{13}C NMR spectrum of compound **4** in DMSO-d_6 (600 MHz)

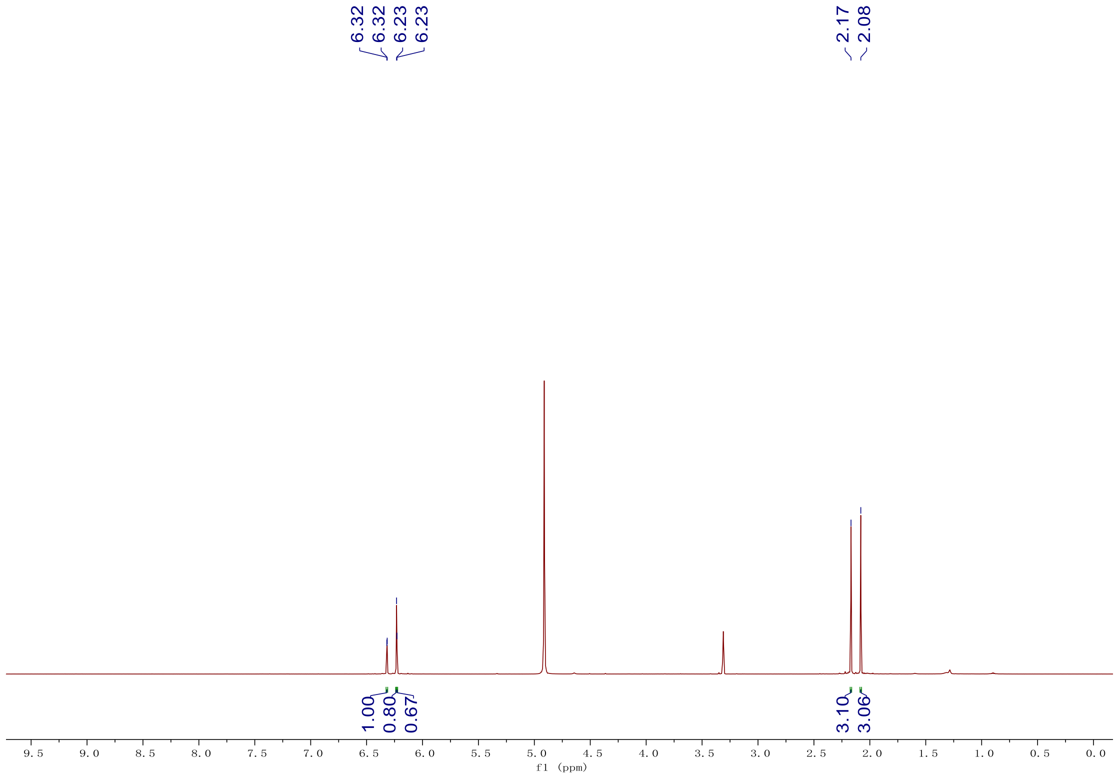

Figure S23. ^{1}H NMR spectrum of compound **5** in CD_3OD (600 MHz)

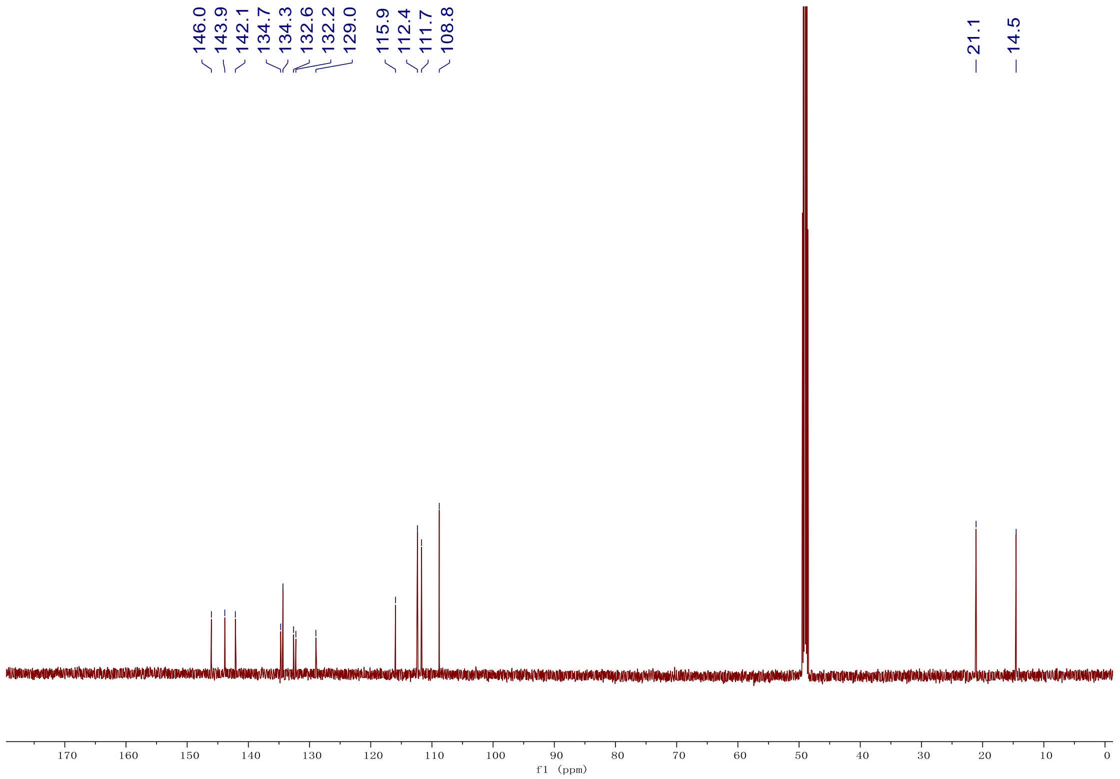

Figure S24. ^{13}C NMR spectrum of compound **5** in CD_3OD (600 MHz)

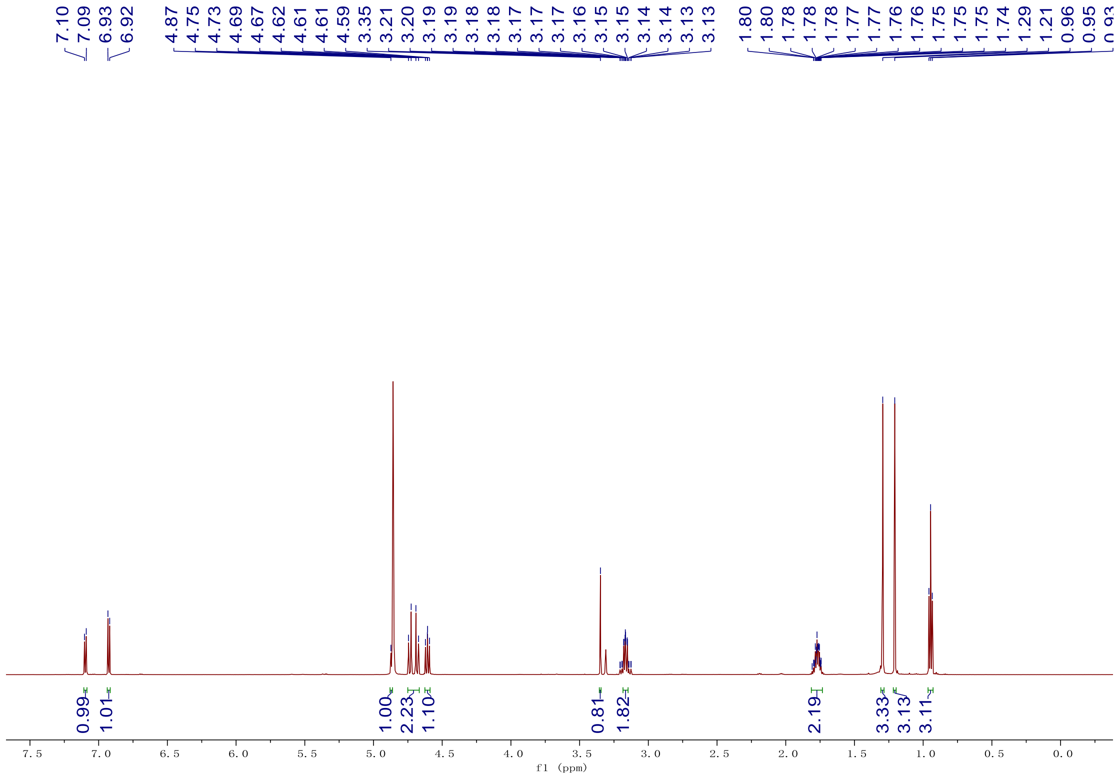

Figure S25. ^{1}H NMR spectrum of compound **6** in CD_3OD (600 MHz)

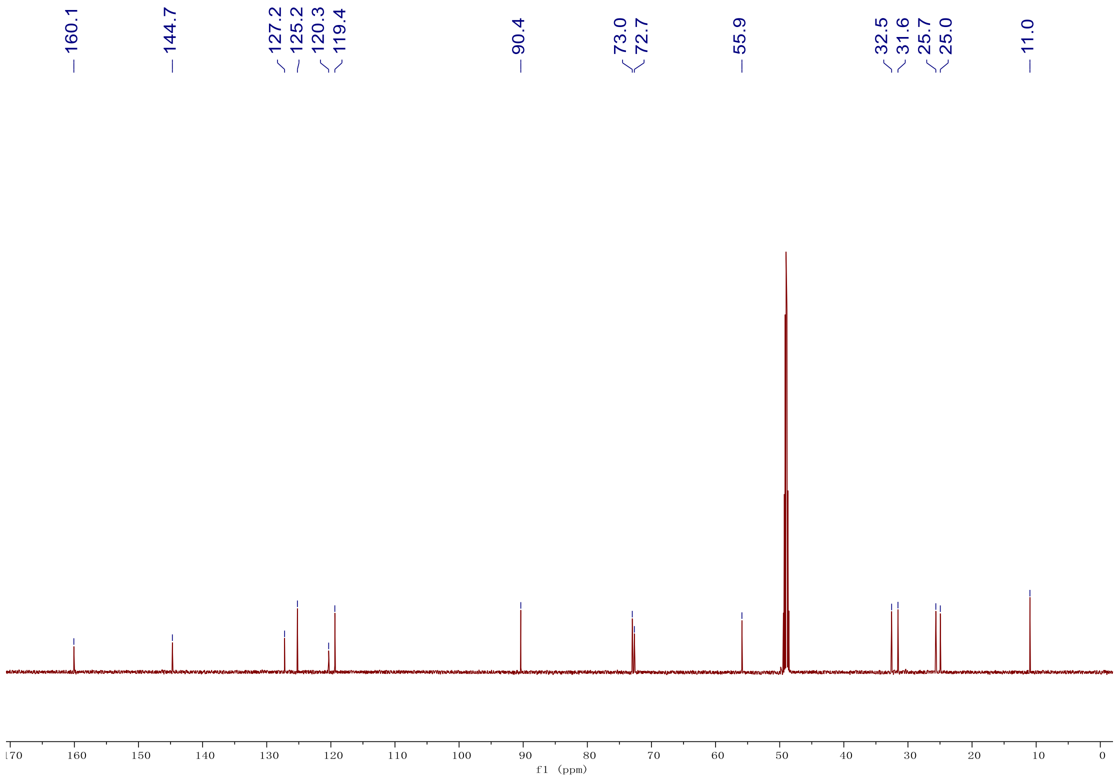

Figure S26. ^{13}C NMR spectrum of compound **6** in CD_3OD (600 MHz)

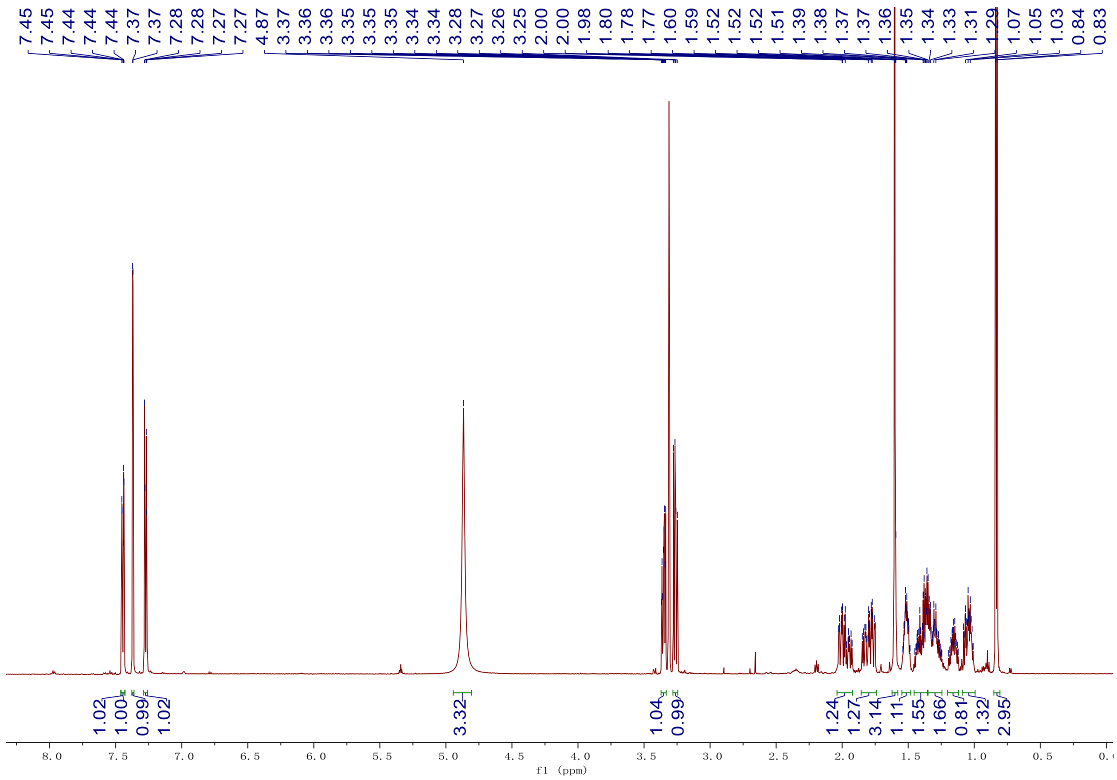

Figure S27. ^{1}H NMR spectrum of compound **7** in CD_3OD (600 MHz)

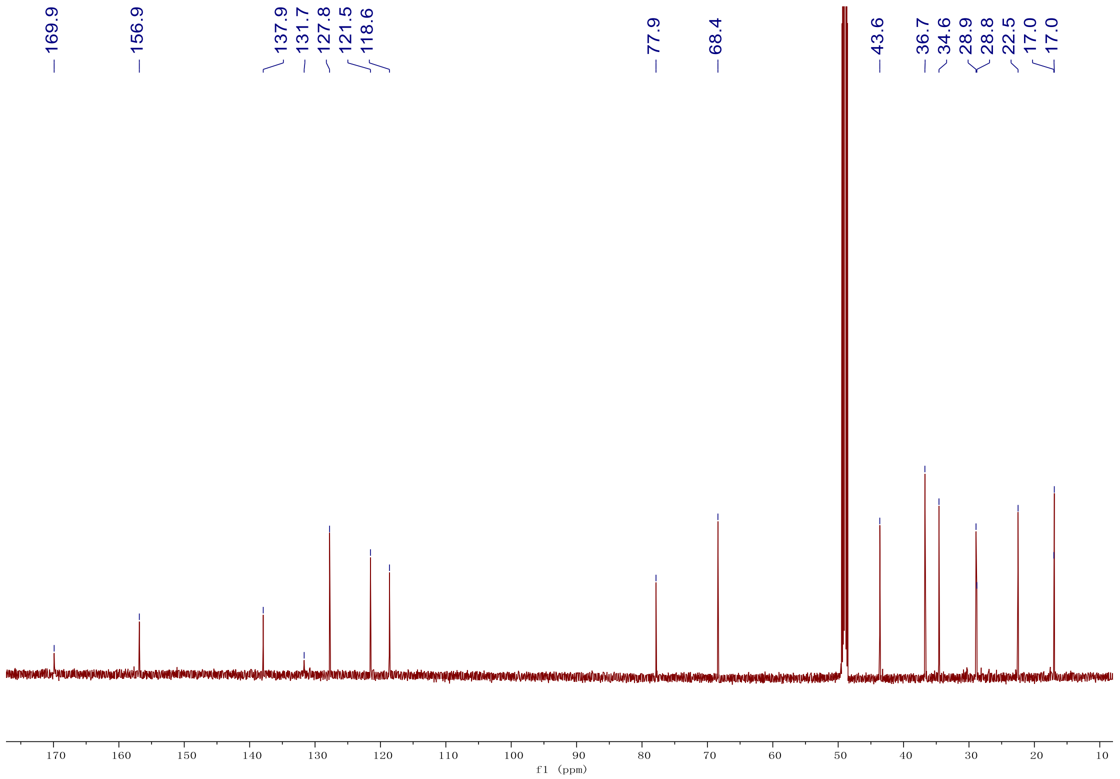

Figure S28. ^{13}C NMR spectrum of compound **7** in CD_3OD (600 MHz)

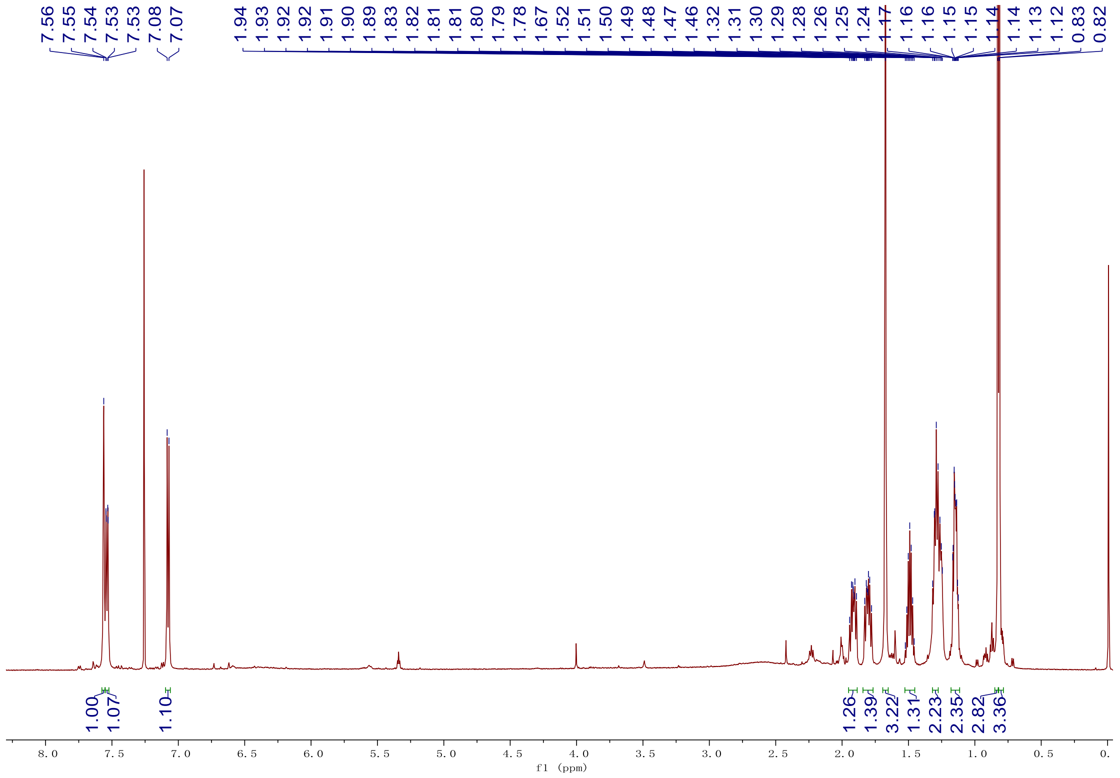

Figure S29. ^{1}H NMR spectrum of compound **8** in $CDCl_3$ (600 MHz)

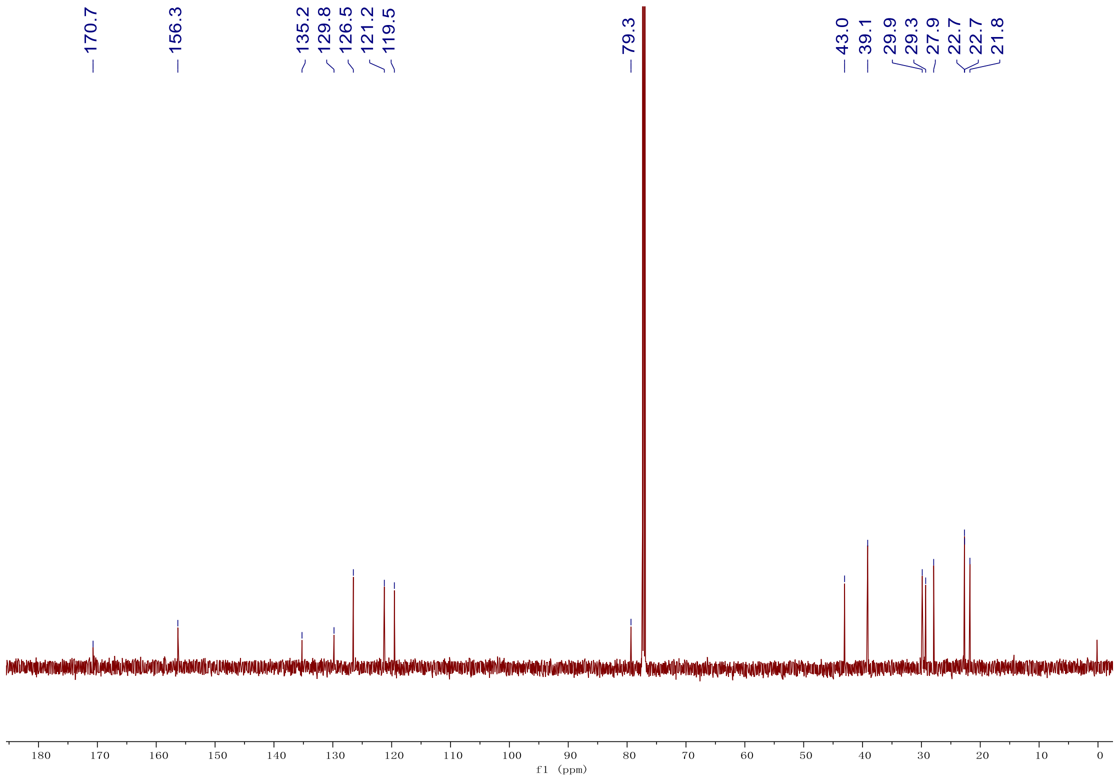

Figure S30. ^{13}C NMR spectrum of compound **8** in $CDCl_3$ (600 MHz)

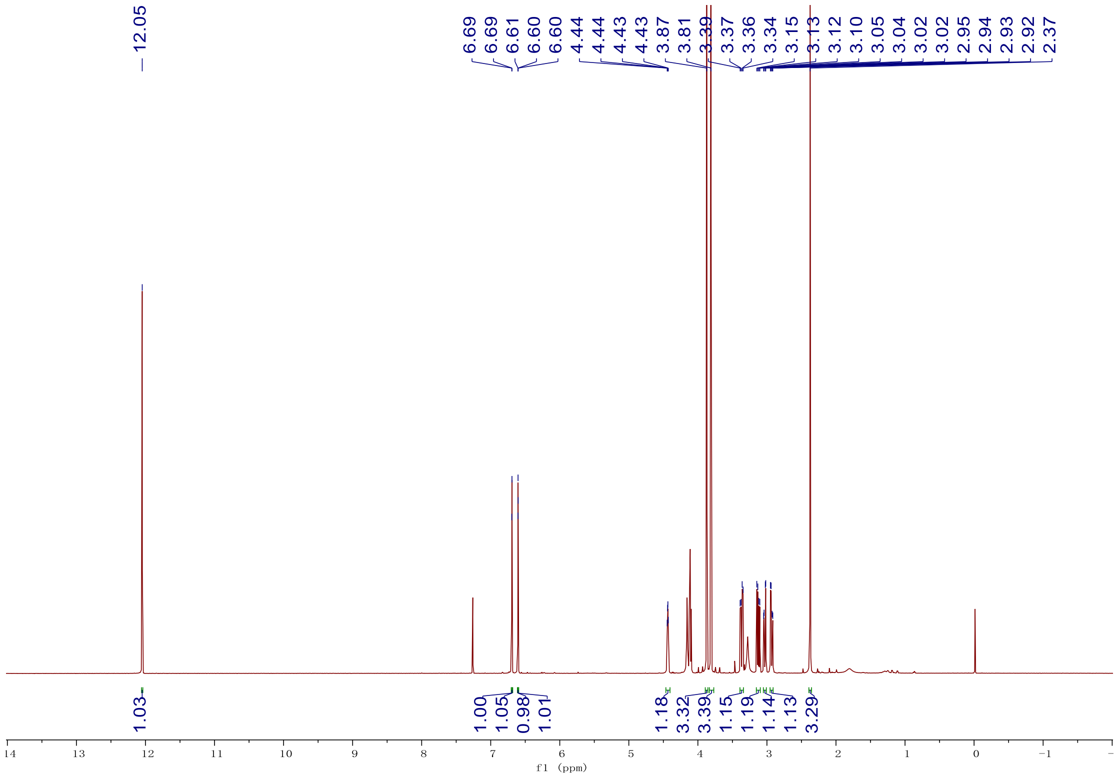

Figure S31. ^{1}H NMR spectrum of compound **9** in $CDCl_3$ (600 MHz)

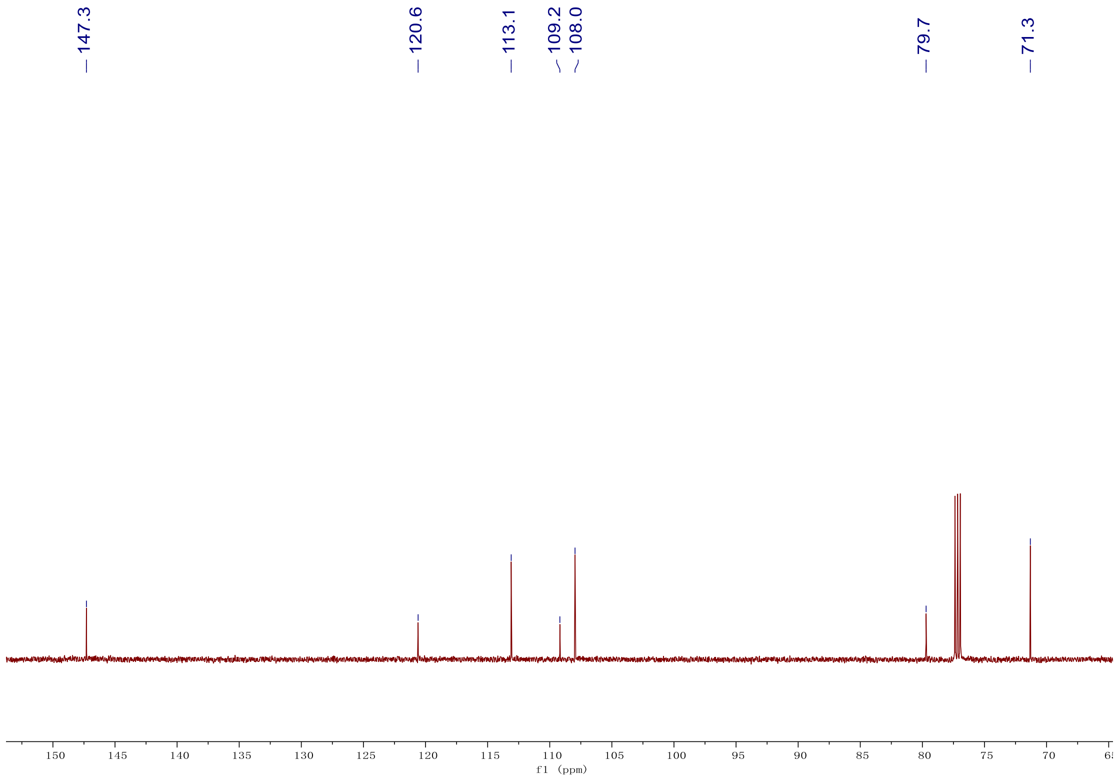

Figure S32. ^{13}C NMR spectrum of compound **9** in $CDCl_3$ (600 MHz)

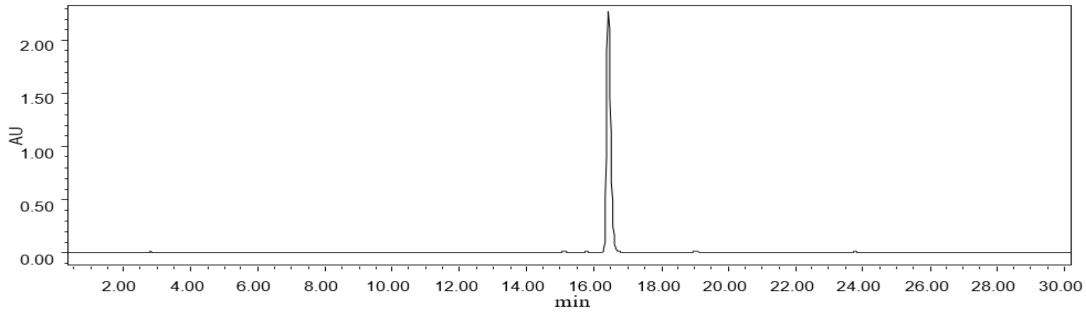

Figure S33. HPLC analysis chromatogram of compound **1**

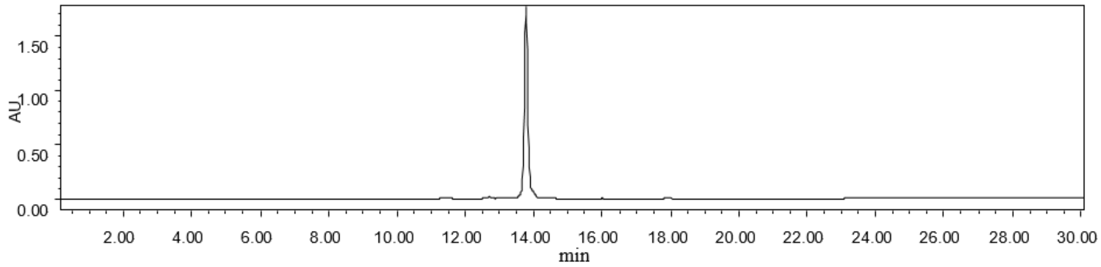

Figure S34. HPLC analysis chromatogram of compound **2**
